# Gradient Graphene Spiral Sponges for Efficient Solar Evaporation and Zero Liquid Discharge Desalination with Directional Salt Crystallization

**DOI:** 10.1002/advs.202400310

**Published:** 2024-03-15

**Authors:** Demin Zhao, Meichun Ding, Tianhao Lin, Zhenying Duan, Rui Wei, Panpan Feng, Jiahui Yu, Chen‐Yang Liu, Chenwei Li

**Affiliations:** ^1^ School of Chemistry and Pharmaceutical Engineering Shandong First Medical University & Shandong Academy of Medical Sciences Jinan 250117 China; ^2^ Science and Technology Innovation Center Shandong First Medical University & Shandong Academy of Medical Sciences Jinan 250117 China; ^3^ CAS Key Laboratory of Engineering Plastics CAS Research/Education Center for Excellence in Molecular Sciences Institute of Chemistry the Chinese Academy of Sciences Beijing 100190 China

**Keywords:** directional salt crystallization, graphene sponge, solar desalination, solar interfacial evaporation, zero liquid discharge

## Abstract

Solar desalination is a promising strategy to utilize solar energy to purify saline water. However, the accumulation of salt on the solar evaporator surface severely reduces light absorption and evaporation performance. Herein, a simple and eco‐friendly method to fabricate a 3D gradient graphene spiral sponge (GGS sponge) is presented that enables high‐rate solar evaporation and zero liquid discharge (ZLD) desalination of high‐salinity brine. The spiral structure of the GGS sponge enhances energy recovery, while the gradient network structures facilitate radial brine transport and directional salt crystallization, which cooperate to endow the sponge with superior solar evaporation (6.5 kg m^−2^ h^−1^ for 20 wt.% brine), efficient salt collection (1.5 kg m^−2^ h^−1^ for 20 wt.% brine), ZLD desalination, and long‐term durability (continuous 144 h in 20 wt.% brine). Moreover, the GGS sponge shows an ultrahigh freshwater production rate of 3.1 kg m^−2^ h^−1^ during the outdoor desalination tests. A continuous desalination–irrigation system based on the GGS sponge for crop growth, which has the potential for self‐sustainable agriculture in remote areas is demonstrated. This work introduces a novel evaporator design and also provides insight into the structural principles for designing next‐generation solar desalination devices that are salt‐tolerant and highly efficient.

## Graphical abstract

A gradient graphene spiral sponge is demonstrated for solar desalination, which has a unique structure for energy recovery, radial brine transport, and directional salt crystallization. It exhibits excellent performance in solar evaporation (6.5 kg m^−2^ h^−1^), salt collection (1.5 kg m^−2^ h^−1^), freshwater production (3.1 kg m^−2^ h^−1^), durability (144 h), and zero liquid discharge desalination. This sponge can potentially enable continuous desalination–irrigation in remote areas for crop growth.

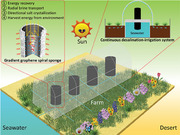

## 1 Introduction

Water scarcity is a global problem that threatens the basic needs of drinking and food production for the growing and developing human population.^[^ ^1^ ^]^ Although water is abundant on Earth, more than 97% of it is seawater.^[^ ^2^ ^]^ Desalination methods such as reverse osmosis and membrane distillation can produce freshwater from seawater, but they often require a lot of energy from nonrenewable energy sources.^[^ ^3^ ^]^ Moreover, these methods face challenges in desalinating high‐salinity brine (>7 wt.%), such as high filtration pressure, energy consumption, and equipment degradation. The resulting high‐salinity brine is discharged into nearby water bodies, causing severe environmental harm.^[^ ^4^ ^]^ To protect the ecosystem and conserve water resources, zero liquid discharge (ZLD) desalination is needed, which can achieve complete water–solute separation.^[^ ^5^, ^6^ ^]^ Therefore, a novel desalination technology that can achieve ZLD with low energy and environmental costs is urgently required. Solar energy is a plentiful and renewable energy source on Earth. Solar interfacial evaporation is a promising technology that uses solar energy to rapidly convert seawater into freshwater with a minimal carbon footprint.^[^ ^7^, ^8^, ^9^ ^]^ As water evaporates, salt crystals gradually form on the evaporator surface, reducing the sunlight absorption and clogging the water and steam channels, thus lowering the evaporation performance.^[^ ^10^ ^]^ Therefore, it is a big challenge to develop a solar evaporator that can efficiently and continuously desalinate high‐salinity brine and achieve ZLD.

Current research on salt‐rejecting properties adopts two main strategies: salt circulation and salt crystallization. The salt circulation strategy aims to prevent salt accumulation on the evaporator surface by rejecting ions,^[^ ^11^ ^]^ designing Janus hierarchical structures,^[^ ^12^, ^13^ ^]^ implementing contactless evaporation,^[^ ^14^ ^]^ or aligning millimeter‐sized channels.^[^ ^15^, ^16^ ^]^ However, this strategy cannot achieve ZLD because some salt ions return to the bulk brine. The salt crystallization strategy offers a more promising solution for ZLD desalination by depositing salt at specific locations, enabling complete separation of solute from water.^[^ ^17^, ^18^, ^19^, ^20^, ^21^ ^]^ This strategy is ideal for brine treatment and wastewater discharge but still faces several severe challenges in practical applications. First, 1D brine transport limits the absorption capacity and significantly reduces the overall evaporation rate. Second, the salt crystallization strategy requires regular removal of salt crystals, which are difficult to fall off automatically from the evaporator surface due to the strong adhesion. If the deposited salt is not removed in time, it will inevitably reduce the evaporation performance. Third, most of the current evaporators are based on porous materials, which have low mechanical strength and make it difficult to meet the high durability and recyclability requirements of solar evaporators. Fourth, many efficient solar evaporators require hazardous or time‐consuming preparation processes, such as high‐pressure, low‐vacuum, or high‐temperature thermal treatments. Therefore, it is challenging to design a robust evaporator via a simple preparation process that can achieve long‐term, highly efficient, and stable evaporation and salt collection for ZLD brine treatment.

**Figure 1:**
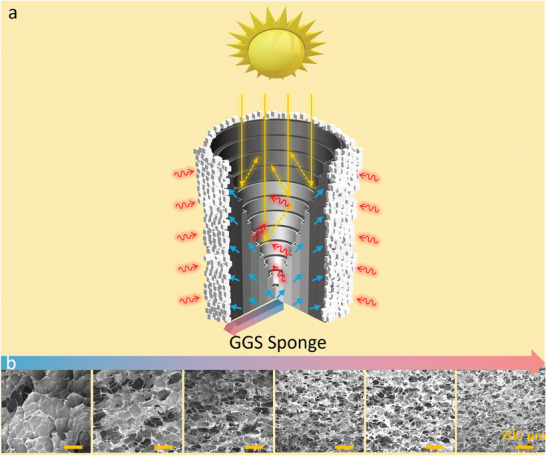
a) A schematic illustration of the GGS sponge as a directional crystallization evaporator. The spiral structure enhances energy recovery, while the gradient graphene network enables radial brine transport and directional salt crystallization. b) SEM images of the gradient graphene network. The pore size of the gradient graphene network gradually decreases from 150 µm at the center to 20 µm at the edge of the sample.

Herein, for the first time, we demonstrate a simple and eco‐friendly method for fabricating a 3D gradient graphene spiral sponge (GGS sponge) that enables high‐rate solar evaporation and ZLD desalination of high‐salinity brine. The method involves air drying and solar reduction, which have advantages over conventional methods such as freeze‐drying, chemical reduction, or thermal reduction. The GGS sponge has a spiral structure and a gradient graphene network with pore sizes ranging from 150 to 20 µm. The spiral structure improves energy recovery, while the gradient graphene network facilitates radial brine transport and directional salt crystallization (**Figure** **1**). As a result, the GGS sponge exhibits an exceptionally high average evaporation rate of 6.5 kg m^−2^ h^−1^ and a high salt collection rate of 1.5 kg m^−2^ h^−1^ in 20 wt.% brine under continuous 1‐sun illumination for 72 h, which is the best comprehensive performance reported so far. The GGS sponge can also achieve complete separation of water and salt from 20 wt.% brine, resulting in ZLD. Moreover, by replacing the outer layer of the GGS sponge, salt can be collected quickly, and the GGS sponge can continuously process high‐salinity brine for up to 144 h. The GGS sponge also exhibits an ultrahigh freshwater production rate of 3.1 kg m^−2^ h^−1^ during the outdoor desalination test. Finally, we present a continuous desalination–irrigation system based on the GGS sponge for wheat growth, which has the potential for self‐sustainable agricultural irrigation in remote areas. This strategy opens up new possibilities for developing high‐performance and low‐cost evaporators for solar desalination and ZLD brine treatment.

## 2 Results and Discussion

### 2.1 Design Principles of the GGS Sponge for Solar Desalination

To meet the requirements of efficient solar energy absorption, energy utilization, salt tolerance, and long‐term stability in a solar desalination evaporator, we developed the solar evaporator based on the following principles: 1) The evaporator needs to possess a robust network structure and chemical stability to ensure long‐term durability and reusability; 2) The fabrication process for the evaporator should be simple and cost‐effective, without requiring expensive equipment; 3) The solar evaporator should not only absorb and convert a wide range of solar energy into heat but also minimize energy loss caused by diffuse reflection and thermal emission, and capture additional energy from the environment; 4) The evaporator should exhibit excellent salt tolerance and sustain high‐rate evaporation for a long time in high‐salinity brine. It should have a unique transport channel that facilitates radial brine transport and promotes directional salt crystallization. Graphene sponges have attracted considerable attention for solar desalination, owing to their low density, high light‐to‐heat conversion efficiency, and chemical stability. We prepared graphene sponges with commercial melamine foam (MF) as the skeleton to achieve the first two requirements. To address the last two requirements, we designed a spiral‐shaped graphene sponge with a gradient network. This design not only enables energy recovery but also facilitates radial brine transport and directional salt crystallization.

### 2.2 Fabrication and Characterization

**Figure 2:**
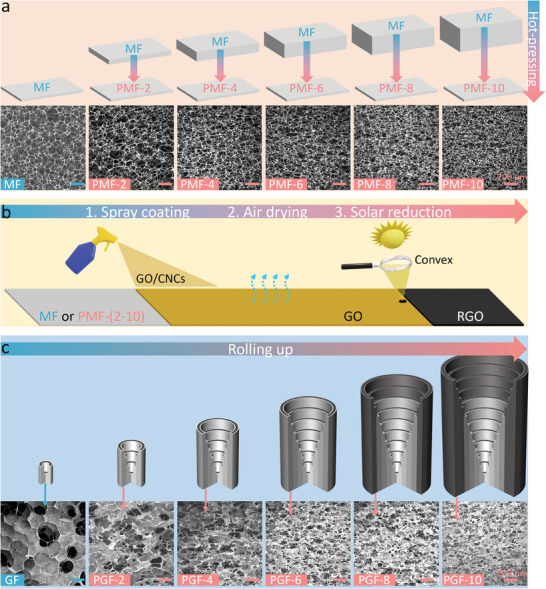
Stepwise fabrication process of the GGS sponge. a) The schematic illustrations for the preparation of PMF‐(2‐10) and the SEM images of MF and PMF‐(2‐10). b) The preparation of GF and PGF‐(2‐10). The MF and PMF‐(2‐10) were continuously transformed into the GGS sponge via three steps: spray coating, air drying, and solar reduction. c) The stepwise fabrication of the GGS sponge and the SEM images of GF and PGF‐(2‐10).

**Figure** **2** illustrates the synthesis process of the GGS sponge. Commercial MF was selected as the skeleton for solar evaporators in this study, because it has favorable characteristics, such as low‐cost, scalable, hydrophilic, highly porous, and thermally insulation.^[^ ^22^ ^]^ MFs with different thicknesses of 2, 4, 6, 8, and 10 mm were hot‐pressed to obtain the pre‐pressed porous melamine films (PMFs) with a thickness of 1 mm, denoted as PMF‐2, PMF‐4, PMF‐6, PMF‐8, and PMF‐10, respectively (Figure 2a). The compression ratios, which represented the ratio of the original thickness of MF to the final thickness of PMF, were 2, 4, 6, 8, and 10, respectively. As shown in Figure S1 (Supporting Information), the densities of PMF‐(2‐10) varied from 7.9 to 78.3 mg cm^−3^ depending on the compression ratio. Scanning electron microscopy (SEM) images showed that MF had a network structure with a pore size of ≈150 µm. With an increase in the compression ratio, the pore size of PMF‐(2‐10) gradually decreased to ≈20 µm, and they exhibited a dense 3D interconnected network structure (Figure 2a). Figure S2 (Supporting Information) showed that PMF‐(2‐10) had superior fluidic transport properties compared to MF as the compression ratio increased. The denser limb network enhanced the capillary force, which resulted in the rapid transport of red ink to the top of PMF.^[^ ^23^ ^]^ Therefore, PMF‐(2‐10) are the ideal skeleton materials for solar evaporators, due to their good water adsorption performance, low density, excellent elasticity, and fatigue resistance properties.^[^ ^23^ ^]^

As shown in Figure 2b, graphene can be easily and eco‐friendly introduced as a photothermal material on MF and PMF‐(2‐10) through three consecutive steps: spraying coating, air drying, and solar reduction. In a typical preparation process, a sheet of MF with a thickness of 1 mm (Figure S3a, Supporting Information) was sprayed with a suspension containing graphene oxide (GO; Figure S4, Supporting Information) and cellulose nanocrystals (CNCs; a hydrophilic additive; Figure S5, Supporting Information). The resulting GO/CNCs/MF composite was directly air dried, avoiding the use of time‐consuming and complex processes, such as freeze–drying or supercritical drying. As shown by the SEM image in Figure S3b (Supporting Information), air drying induced the self‐assembly of GO sheets on the MF skeleton, forming a 3D network structure. This result demonstrates that the MF network effectively prevented excessive GO sheet stacking due to capillary forces during air drying.^[^ ^24^, ^25^ ^]^ The GO/CNCs/MF composite was subsequently subjected to focused sunlight irradiation, resulting in a rapid color change from brown to black, indicating the reduction of GO to reduced graphene oxide (RGO) (Figure S6, Supporting Information). Figure S3c (Supporting Information) shows that RGO/CNCs/MF (GF) maintained its 3D network structure after solar reduction, and the surfaces of RGO sheets showed some wrinkles. X‐ray photoelectron spectroscopy (XPS), X‐ray diffraction (XRD), and Raman analysis confirmed the removal of most oxygen‐containing functional groups from GO during the solar reduction process, indicating the effective reduction of GO to RGO (Figures S7–S9, Supporting Information). This method also enabled the fabrication of highly porous and interconnected 3D graphene networks from the PMF‐(2‐10) with a thickness of 1 mm (Figure 2b). The gradient graphene spiral sponge (GGS sponge) was fabricated by sequential rolling of GF and RGO/CNCs/PMF‐(2‐10) (PGF‐(2‐10)) composites with different widths and pore sizes. The GGS sponge exhibited a pore size gradient from ≈150 to ≈20 µm (Figure 2c) and a porosity gradient from 98.4% to 80.3% (Table S1, Supporting Information).

### 2.3 Mechanical Properties

The mechanical properties of the solar evaporator are essential for achieving long‐term, efficient, and stable solar desalination. Herein, we systematically investigated the mechanical properties of GF and PGF‐(2‐10) under various modes, such as compression, bending, and tension. Figure S10 (Supporting Information) shows the compression tests of GF and PGF‐(2‐10) with a strain of 80%. The compressive stress of PGF‐(2‐10) ranged from 0.087 to 0.449 MPa, which is 1.7–9.0 times higher than that of GF, respectively. The GF and PGF‐(2‐10) maintained their bending modulus and bending strength at almost 100% after the force was released, indicating excellent bending performance (Figure S11, Supporting Information). As shown in Figure S12 (Supporting Information), the fracture strength and breaking elongation of the GF and PGF‐(2‐10) ranged from 0.101 to 1.360 MPa and from 18.1% to 29.9%, respectively. To assess the robustness and durability of GF and PGF‐(2‐10) under real conditions, various extreme tests were performed (Figure S13, Supporting Information). The GF and PGF‐(2‐10) were exposed to different harsh environments, such as 20 cycles of bending in air and water, acid (pH ≈1, 24 h), base (pH ≈14, 24 h), high temperature (≈95 °C, 1 h), and ultrasonic agitation (400 W, 1 h). The GF and PGF‐(2‐10) maintained their appearances without noticeable changes after these tests, demonstrating their outstanding durability and structural stability. The outstanding elasticity, flexibility, and durability of GF and PGF‐(2‐10) enable the GGS sponge to achieve long‐term and stable solar desalination performance.

### 2.4 Directional Salt Crystallization Enabled by the Gradient Graphene Network

**Figure 3:**
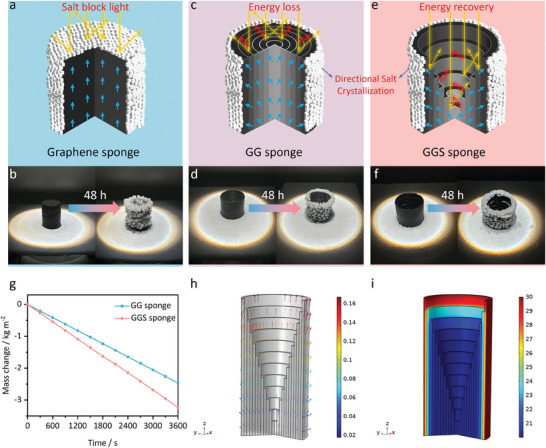
a) Schematic showing salt crystallization on the surface of the graphene sponge. b) Photographs showing salt crystals formed on the graphene sponge in 20 wt.% brine under one sun illumination for 48 h. c) Schematic illustration of the directional salt crystallization of the GG sponge. d) Photographs of the GG sponge during a 48 h desalination test in 20 wt.% brine under 1‐sun illumination. e) Schematic illustration of the directional salt crystallization and energy recovery of the GGS sponge. f) Photographs of the GGS sponge during a 48 h desalination test in 20 wt.% brine under 1‐sun illumination. g) The mass change of 20 wt.% brine versus time with the GG and the GGS sponges under one sun illumination. Numerical simulation of h) fluid velocity and i) salinity distributions in the GGS sponge in 20 wt.% brine under 1‐sun illumination.

Salt accumulation on the interface is a major challenge for solar interface evaporators that treat concentrated brine.^[^ ^26^, ^27^ ^]^ As water evaporates from the interface, the local salt concentration increases and eventually exceeds saturation, leading to salt crystallization on the interface. This impedes light absorption and water transport channels, resulting in the reduction of evaporation performance (**Figure** **3**a). Herein, the solar desalination tests were performed using an artificial brine solution with 20 wt.% NaCl. Figure 3b shows that the graphene sponge with random pores suffered from severe salt accumulation, due to its low water transfer efficiency (Figure S14, Supporting Information).^[^ ^28^ ^]^ To solve the salt accumulation problem, we designed a gradient graphene sponge (GG sponge), as shown in Figure S15 (Supporting Information). The gradient graphene network induced a radial brine transport effect by generating a capillary force difference along the pore size gradient (Figure 3c). As vapor was continuously generated, the radial brine flow enhanced the brine concentration on the outer surface of the GG sponge, leading to directional salt crystallization (Figure 3d; Figure S16, Supporting Information).

### 2.5 Energy Recovery and Directional Salt Crystallization Enabled by the Gradient Graphene Spiral Network

However, the 2D upper surface of the GG sponge caused energy loss due to diffuse reflection and thermal radiation, which limited its evaporation performance. To overcome this limitation, we further optimized the design of the GG sponge and developed a gradient graphene spiral sponge (GGS sponge), which achieved both energy recovery^[^ ^7^, ^10^, ^18^ ^]^ and directional salt crystallization, as shown in Figure 3e,f and Figure S17 (Supporting Information). To evaluate the energy recovery effect of the spiral structure, we measured the light absorption properties of the GG and the GGS sponges using UV–vis–NIR spectroscopy with an integrating sphere from 300 to 2500 nm. As shown in Figure S18 (Supporting Information), the GGS sponge had a higher absorbance and a lower reflectance than the GG sponge. Figure S19 (Supporting Information) shows that after evaporating for 15 min, the centers of the top surfaces of the GG and GGS sponges reached stable temperatures of 41.3 and 37.5 °C, respectively. Based on the heat loss calculation (Section 4.3 Thermal Loss Analysis, Supporting Information), the total heat loss (conduction + convection + radiation) of the GG and the GGS sponges were 19.2% and 6.9% of the input solar heat flux (1 kW m^−2^), respectively. Therefore, the GGS sponge exhibited a higher evaporation rate of 3.3 kg m^−2^ h^−1^, whereas the GG sponge had a rate of 2.5 kg m^−2^ h^−1^ (Figure 3g). Moreover, the evaporation process of the GGS sponge under one sun illumination was simulated using COMSOL Multiphysics software to reveal the critical role of the gradient graphene spiral network in directional salt crystallization. The simulation included the brine flow and salinity distributions. Figure 3h shows that the gradient network structures generated a capillary force difference that induced a radial brine flow from the center to the outer surface. We performed a steady‐state simulation of the salinity distributions of the GGS sponge with an input salinity of 20 wt.% (Figure 3i). The results showed that only the outer surface of the GGS sponge had a salinity exceeding the saturation concentration, while the inner surface had a salinity almost unchanged from the initial brine concentration. This indicated that salt crystallization occurred selectively on the outer surface of the GGS sponge. Therefore, the radial brine transport enabled by the gradient graphene spiral network led to directional salt crystallization on the outer surface. Moreover, the concave middle region of the GGS sponge remained highly humid owing to its proximity to the water source, which also prevented salt crystallization in this area.^[^ ^8^ ^]^

### 2.6 Continuous and High‐Rate Solar Desalination with ZLD Brine Treatment

**Figure 4:**
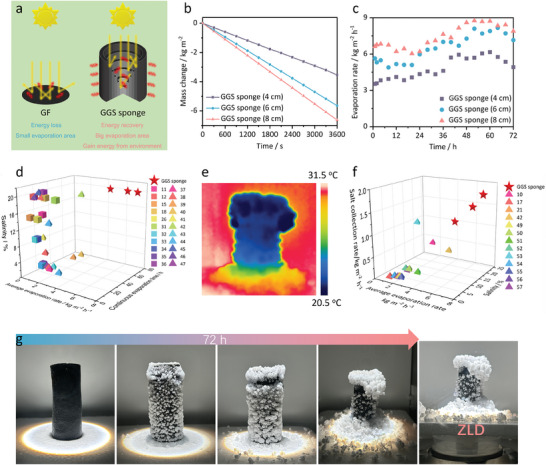
a) A comparison of the evaporation area and the environmental energy harvesting of 2D GF and 3D GGS sponge. b) The mass change of 20 wt.% brine versus time with the GGS sponge (4, 6, and 8 cm) under 1‐sun illumination. c) The evaporation rates of the GGS sponges (4, 6, and 8 cm) in 20 wt.% brine under 1‐sun illumination for continuous 72 h. d) A comparison of the evaporation rates and continuous evaporation times of the GGS sponges and other reported solar desalination devices under 1‐sun illumination at maximum salinity. e) Infrared radiation thermal image of the salt layer on the outer surface of the GGS sponge (6 cm). f) Salt collection rate of the GGS sponges compared with other solar desalination devices. g) The photographs of the GGS sponge (6 cm) during a continuous 72 h desalination test under 1‐sun illumination.

Solar interfacial evaporation is governed by three energy fluxes: the solar energy input, the vaporization energy output, and the energy loss to the environment.^[^ ^29^ ^]^ Therefore, traditional 2D solar evaporators cannot achieve the ideal 100% solar‐to‐vapor conversion efficiency due to the energy dissipation through heat exchange with the surroundings. However, 3D solar evaporators can break the theoretical limit of solar‐to‐vapor conversion efficiency.^[^ ^30^ ^]^ The side surface of the GGS sponge has low solar absorption, and the evaporation from this surface produces an evaporative cooling effect that lowers the temperature of the side surface below the ambient temperature (Figure S19b, Supporting Information).^[^ ^31^ ^]^ As a result, the increased height of the 3D structure can enlarge the effective evaporation area by harvesting more energy from the environment (**Figure** **4**a). To study the effect of sample height on solar desalination, GGS sponges with different heights (4, 6, and 8 cm) were fabricated. The solar evaporation rate of the GGS sponge with varying heights was assessed by an electrical balance under simulated illumination (Figure S20, Supporting Information). As shown in Figure 4b, the evaporation rate of GGS sponges increased with their height, with values of 3.5, 5.6, and 6.6 kg m^−2^ h^−1^ for sponges of 4, 6, and 8 cm, respectively. We also assessed the solar desalination performance of GGS sponges (4, 6, and 8 cm) by continuously desalinating 20 wt.% brine for 72 h under 1‐sun illumination. As shown in Figure 4c, the average evaporation rates of GGS sponges (4, 6, and 8 cm) were 4.7, 6.5, and 7.5 kg m^−2^ h^−1^, respectively. The GGS sponges outperformed other reported solar desalination devices^[^ ^11^, ^12^, ^15^, ^18^, ^26^, ^31^, ^32^, ^33^, ^34^, ^35^, ^36^, ^37^, ^38^, ^39^, ^40^, ^41^, ^42^, ^43^, ^44^, ^45^, ^46^, ^47^ ^]^ in terms of evaporation rate, evaporation time, and salinity (Figure 4d; Table S2, Supporting Information).

As shown in Figure S21 (Supporting Information), the GGS sponges had a gradient network structure that enabled radial brine transport, resulting in spontaneous salt deposition on the outer surface rather than the inner surface. This ensured sufficient evaporation area on the inner surface of the GGS sponges for efficient and stable solar evaporation. Moreover, the GGS sponges utilized their entire outer surface for salt crystallization, which offered them a much larger crystallization area than other directional crystallization evaporators (Figure S22, Supporting Information). The salt deposition was composed of loosely packed NaCl crystals with abundant void space, which facilitated brine transport and vapor release.^[^ ^27^, ^48^ ^]^ Due to the evaporative cooling effect and limited solar absorption, the surface temperature of the salt layer on the GGS sponge was lower than the ambient temperature (Figure 4e). As water evaporated, the growing salt layer harvested more energy from the environment and increased the effective evaporation area (Figure S23, Supporting Information). The gradual increase of effective evaporation area enhanced the evaporation rates of GGS sponges. Therefore, after desalinating continuously for 24 h, the evaporation rate of GGS sponges (4, 6, and 8 cm) increased over time and eventually reached 6.2, 8.2, and 8.7 kg m^−2^ h^−1^, respectively (Figure 4c).

Most solar desalination devices face the problem of salt accumulation, which prevents them from achieving ZLD and causes environmental damage from the release of concentrated waste brine. In contrast, GGS sponges can overcome this challenge and achieve complete separation of water and salt from 20 wt.% brine in a continuous 72 h desalination process, resulting in ZLD (Figure 4g). The salts deposited on the outer surface of the GGS sponge could be easily removed and collected with a stainless‐steel medicine spoon after the test (Figure S24a, Supporting Information). After the removal, the inner surface of the GGS sponge was almost free of salt (Figure S24b, Supporting Information). The salt collection rate of the GGS sponge was determined by dividing the mass of collected salt (Figure S24c, Supporting Information) by the product of the evaporation time and the projected area. The GGS sponges (4, 6, and 8 cm) achieved high salt collection rates of 1.1, 1.5, and 1.8 kg m^−2^ h^−1^, respectively, which surpassed those of other reported solar desalination devices^[^ ^10^, ^17^, ^21^, ^42^, ^49^, ^50^, ^51^, ^52^, ^53^, ^54^, ^55^, ^56^, ^57^ ^]^ (Figure 4f; Table S3, Supporting Information). This demonstrates the outstanding performance of the GGS sponges in achieving both efficient salt collection and solar evaporation. The results indicate that the GGS sponge (6 cm) achieved the best balance between performance and economy. Therefore, we chose the GGS sponge (6 cm) as the main research object for this study, unless otherwise stated.

### 2.7 Solar Desalination Under Natural Sunlight Conditions and the Continuous Desalination–Irrigation System

**Figure 5:**
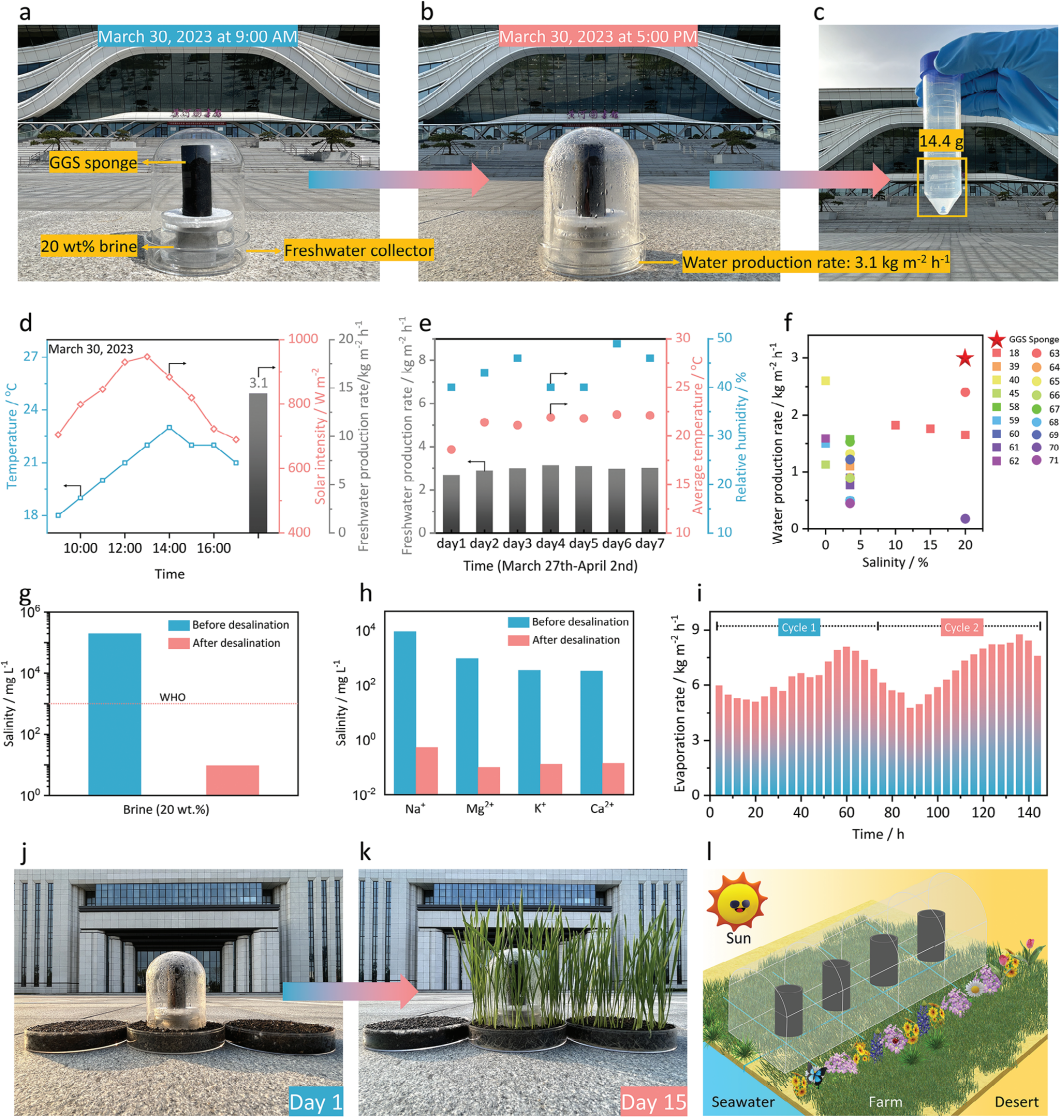
Photographs of the homemade device for freshwater collection based on the GGS sponge, before a) and after b) outdoor solar desalination on March 30, 2023. c) Photograph of the freshwater collected from 9:00 AM to 5:00 PM. d) The environmental conditions (temperature and solar irradiation intensity) during the outdoor solar desalination test. e) The freshwater production rate and conditions (average temperature and relative humidity) during the outdoor solar desalination tests from March 27 to April 2. f) The freshwater production performance of the outdoor solar desalination device based on the GGS sponge in 20 wt.% brine, compared with recent literature reports. g) The measured concentration of Na^+^ in 20 wt.% brine before and after solar desalination. h) The measured concentrations of primary ions in a real seawater sample (from Qingdao, China) before and after solar desalination. i) The evaporation rates of the GGS sponge in 20 wt.% brine under one sun illumination for 144 h. After collecting the salt layer at the end of the first cycle test, the GGS sponge could resume a second cycle of continuous 72 h test. j) The homemade set‐up for the continuous desalination–irrigation system. k) Photograph of wheat growth situation irrigated by 20 wt.% brine, purified water, and tap water, respectively, after 15 days. l) Sketch of potential applications of the continuous desalination–irrigation system.

Solar desalination devices have faced challenges with low water production rates, even with the significant increase in evaporation performance. The primary objective of solar desalination is to enhance efficient freshwater production. To evaluate the freshwater productivity of the GGS sponge under natural environmental conditions, we constructed and tested a solar desalination device on the campus of Shandong First Medical University (Jinan, China) from March 27 to April 2, 2023. As shown in **Figure** **5**a, a GGS sponge, with a diameter of 2.7 cm and a height of 6 cm, was immersed in a container filled with 20 wt.% brine. The container was sealed with a transparent glass cover. As shown in Figure 5d, we conducted the continuous solar desalination test from 9:00 AM to 5:00 PM under natural sunlight with an average light intensity of ≈ 0.8 kW m^−2^. The maximum values of natural light intensity (0.9 kW m^−2^) and ambient temperature (23.0 °C) were recorded at 1:00 PM and 2:00 PM, respectively. As the GGS sponge evaporated, it caused water vapor to condense on the inner surface of the glass cover, forming freshwater droplets that continuously flowed into the container for collection (Figure 5b and Figure S25, Supporting Information). As shown in Figure 5c, the GGS sponge evaporator produced 14.4 g of freshwater after 8 h of sunlight radiation, corresponding to a production rate of ≈3.1 kg m^−2^ h^−1^. To assess the stability of the GGS sponge for long‐term outdoor desalination tests, we measured and analyzed the freshwater production rates, temperatures, light intensity, and relative humidity from March 27 to April 2, 2023 (Figure 5e; Figure S26, Supporting Information). Over a week, the GGS sponge‐based desalination device produced freshwater at high and stable rates of 2.7–3.1 kg m^−2^ h^−1^, exceeding all previously reported single‐stage solar desalination devices^[^ ^18^, ^39^, ^40^, ^45^, ^58^, ^59^, ^60^, ^61^, ^62^, ^63^, ^64^, ^65^, ^66^, ^67^, ^68^, ^69^, ^70^, ^71^ ^]^ (Figure 5f and Table S4, Supporting Information). This demonstrates the outstanding performance of the GGS sponge in achieving efficient freshwater production under natural sunlight conditions.

To evaluate the effect of solar desalination, we measured the ion concentrations in the freshwater obtained from the process. As shown in Figure 5g, the concentration of Na^+^ in the freshwater condensed from 20 wt.% brine by GGS sponge was significantly lower than the drinking water standard defined by the World Health Organization (WHO). In addition, we tested real seawater (from the Yellow Sea, Qingdao, China) for solar desalination using the GGS sponge. The results indicated a remarkable reduction in the primary ions in the condensed water, with an ion rejection rate of above 99.9% (Figure 5h). For practical applications, reusability and durability are crucial factors for solar desalination devices. To enable long‐term solar desalination, fast salt removal after each cycle test is essential. However, most evaporators can only shed the deposited salts automatically under specific conditions, such as pre‐wetting of the evaporation interface,^[^ ^17^ ^]^ low‐rate evaporation in the dark,^[^ ^19^ ^]^ or excessive salt crystals.^[^ ^49^ ^]^ Designing an ideal solar desalination device that combines efficient and stable evaporation with rapid salt collection is a big challenge. To address this challenge, we replaced the outer PGF‐10 of the GGS sponge for quick salt collection after each cycle desalination test (Figure S27, Supporting Information). To evaluate the durability and reusability of the GGS sponge, we used it to treat 20 wt.% brine under 1‐sun illumination for continuous 144 h (Figure 5i). The GGS sponge maintained a high average evaporation rate (6.3 kg m^−2^ h^−1^) and achieved ZLD desalination in the first cycle of 72 h. After removing the deposited salt (salt collection rate: 1.4 kg m^−2^ h^−1^) by simply replacing the outer PGF‐10 layer, the GGS sponge could resume a second cycle test within 1 min (Movie S1, Supporting Information). The GGS sponge exhibited a similar excellent evaporation performance (6.8 kg m^−2^ h^−1^) and salt collection rate (1.5 kg m^−2^ h^−1^) in the second cycle test, demonstrating its excellent reusability and long‐term durability, which is the best result reported so far for continuous solar desalination of high‐salinity brine (Table S5, Supporting Information). To demonstrate the practical application of the GGS sponge, we evaluated its cost‐effectiveness as an evaporator (Table S6, Supporting Information). The GGS sponge achieved an outstanding average evaporation rate of 6.6 kg m^−2^ h^−1^ and a cost‐effectiveness of 122.52 g h^−1^ $^−1^, surpassing those of the previously reported evaporators^[^ ^27^, ^72^, ^73^, ^74^, ^75^, ^76^, ^77^ ^]^ (Figure S28, Supporting Information).

Some remote areas have limited freshwater resources for drinking and irrigation because of their poor infrastructure. Seawater, which is abundant but unsuitable for seed germination due to its high salinity, inhibits crop growth. To address this issue, we propose a solar desalination device based on the GGS sponge, which can produce freshwater from high‐salinity brine efficiently under natural sunlight (Figure 5j). This miniature desalination–irrigation device consists of four components: 1) the GGS sponge, 2) a tank with 20 wt.% brine, 3) a glass cover for condensation, and 4) soil (Figure S29, Supporting Information). The GGS sponge enables the evaporation of brine, and the water vapor is condensed on the glass cover and then flows into the soil to support wheat growth. We observed that wheat irrigated with purified water exhibited a similar growth rate to the wheat irrigated with tap water, reaching ≈135 mm in 15 days (Figure 5k; Figure S30, Supporting Information). In contrast, wheat seeds sown in brine failed to germinate (Figure S31, Supporting Information). These results illustrate the potential of our solar desalination device in agricultural irrigation for remote areas (Figure 5l).

## 3 Conclusion

We have fabricated a GGS sponge with a spiral structure and gradient network structures by a simple and eco‐friendly method for high‐rate solar evaporation and ZLD desalination of high‐salinity brine. The novel design not only enhances energy recovery but also facilitates radial brine transport and directional salt crystallization. As a result, the GGS sponge exhibits an average evaporation rate of 6.5 kg m^−2^ h^−1^ and a salt collection rate of 1.5 kg m^−2^ h^−1^ in 20 wt.% brine under 1‐sun illumination for 72 h, which is the best comprehensive performance reported so far. The GGS sponge can also achieve complete separation of water and salt from 20 wt.% brine, resulting in ZLD. Moreover, the GGS sponge also exhibits an ultrahigh freshwater production rate of 3.1 kg m^−2^ h^−1^ during the outdoor desalination test. The purified water can be used for crop growth through a solar‐driven continuous desalination–irrigation system, which boosts the practical application of the solar desalination device and advances the field of self‐sustainable agricultural irrigation.

## Conflict of Interest

The authors declare no conflict of interest.

## Supporting information

Supporting Information

Supplemental Movie 1

## Data Availability

The data that support the findings of this study are available from the corresponding author upon reasonable request.
