# Supplementary material for: Gradient Graphene Spiral Sponges for Efficient Solar Evaporation and Zero Liquid Discharge Desalination with Directional Salt Crystallization
Source: Adv Sci (Weinh). 2024 Mar 15;11(22):2400310. doi: 10.1002/advs.202400310 (PMC11165548; doi:10.1002/advs.202400310)
Supplement: Supplementary file 1 — Supporting Information [file ADVS-11-2400310-s001.pdf]

ADVANCED SCIENCE
Open Access

Supporting Information

for *Adv. Sci.*, DOI 10.1002/advs.202400310

Gradient Graphene Spiral Sponges for Efficient Solar Evaporation and Zero Liquid Discharge Desalination with Directional Salt Crystallization

Demin Zhao, Meichun Ding, Tianhao Lin, Zhenying Duan, Rui Wei, Panpan Feng, Jiahui Yu, Chen-Yang Liu and Chenwei Li**

Supporting Information

Gradient Graphene Spiral Sponges for Efficient Solar Evaporation and Zero Liquid Discharge Desalination with Directional Salt Crystallization

Demin Zhao,[ab] Meichun Ding,[ab] Tianhao Lin,[a] Zhenying Duan,[ab] Rui Wei,[ab] Panpan Feng,[ab] Jiahui Yu,[b] Chen-Yang Liu,[c] Chenwei Li*[ab]*

[a] School of Chemistry and Pharmaceutical Engineering, Shandong First Medical University & Shandong Academy of Medical Sciences, Jinan 250117, China

[b] Science and Technology Innovation Center, Shandong First Medical University & Shandong Academy of Medical Sciences, Jinan 250117, China

[c] CAS Key Laboratory of Engineering Plastics, CAS Research/Education Center for Excellence in Molecular Sciences, Institute of Chemistry, the Chinese Academy of Sciences, Beijing 100190, China

* Corresponding Author: mcding@iccas.ac.cn (Meichun Ding); lichenwei@iccas.ac.cn (Chenwei Li)

1. Supplementary Figures and Tables

1.1 The Densities of MF and PMF-(2-10)

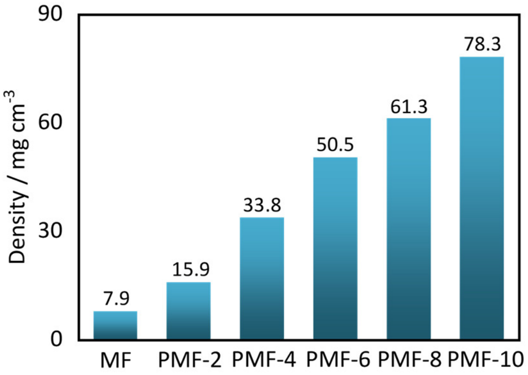

Figure S1. The densities of MF and PMF-(2-10). The density of PMF-(2-10) increased gradually with the increase in the compression ratios.

1.2 The Fluidic Transport Properties of MF and PMF-(2-10)

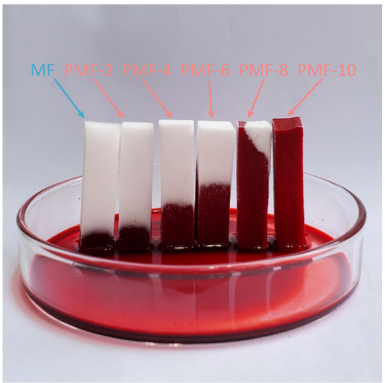

Figure S2. The photographs of MF and PMF-(2-10) after red ink adsorption. PMF-(2-10) exhibited superior fluidic transport properties compared to MF with the increasing in compression ratio.

1.3 SEM Images of MF, GO/CNCs/MF, and GF

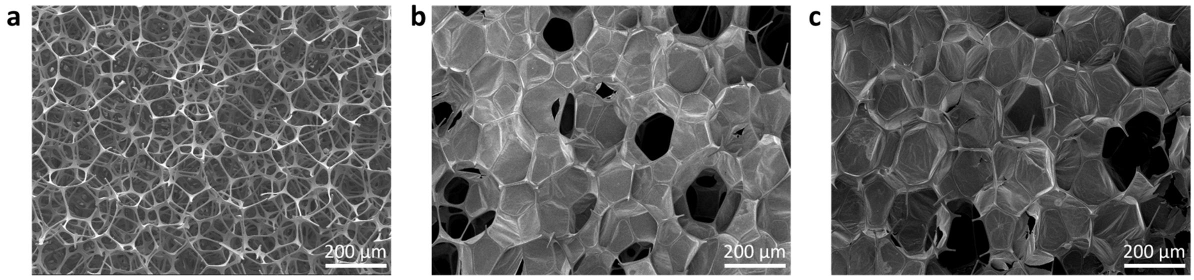

Figure S3. SEM images of (a) MF, (b) GO/CNCs/MF, and (c) GF.

1.4 Atomic Force Microscopy (AFM) Image of GO

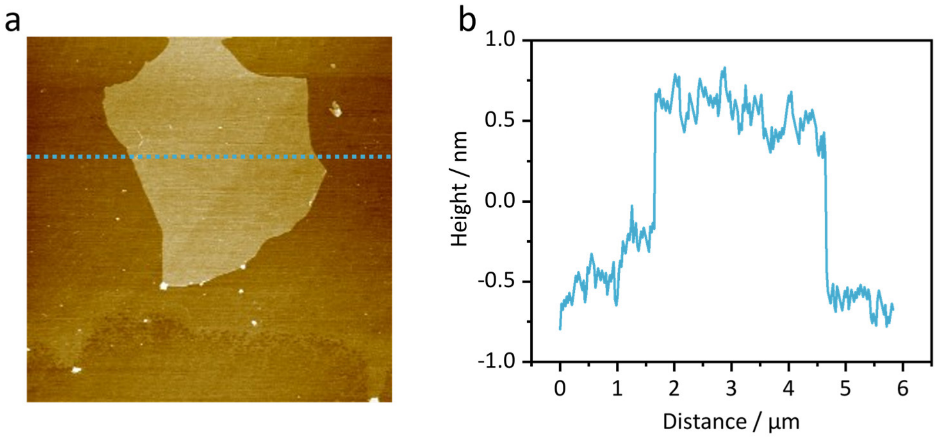

Figure S4. The AFM image (a) and its corresponding height profile (the blue dotted line); (b) show that the GO sheets are monolayers with a thickness of ~ 0.8 nm.

1.5 HRTEM Image of CNCs

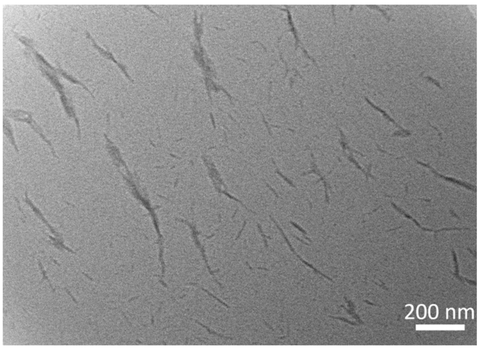

Figure S5. HRTEM Image of CNCs.

1.6 Solar Reduction of GO

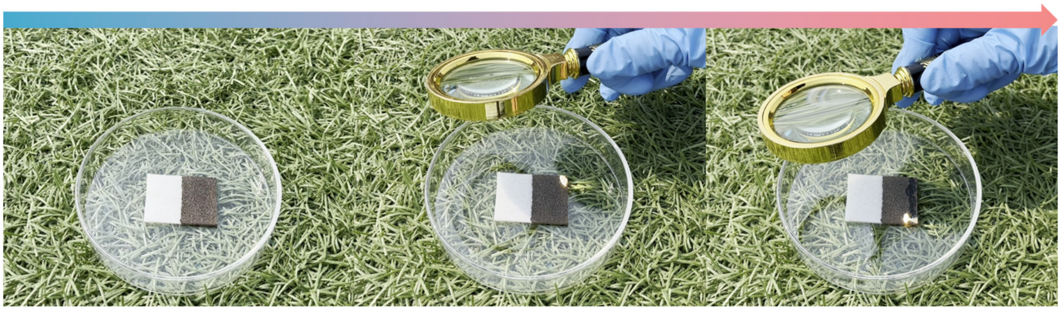

Figure S6. The Photographs of solar reduction of GO/CNCs/MF composite by using a magnifying glass. The brown color turned to black, indicating the reduction of GO to RGO.

1.7 XPS Analysis of GO and RGO

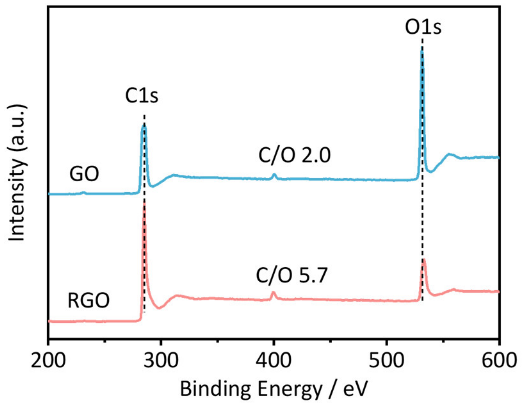

Figure S7. XPS spectra of GO and RGO. The C/O mole ratio of rGO increased from 2.0 in GO to 5.7, which shows that most oxygen-containing functional groups were eliminated during the solar reduction process.

1.8 XRD Analysis of GO and RGO

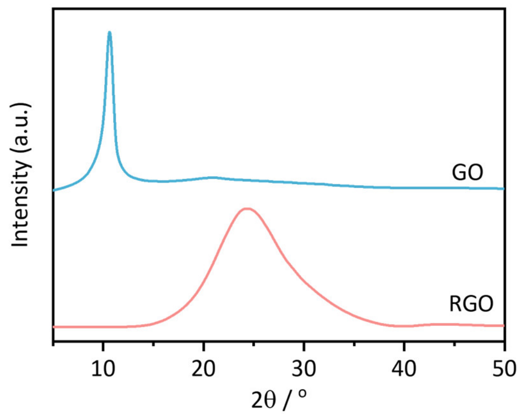

Figure S8. XRD spectra of GO and RGO. The XRD peak of RGO shifted from 10.6° to 24.4°, indicating the removal of oxygen-containing functional groups (such as C-COOH, C-OH, C-O-C, and C=O) from the GO sheets during the solar reduction process.

1.9 Raman Analysis of GO and RGO

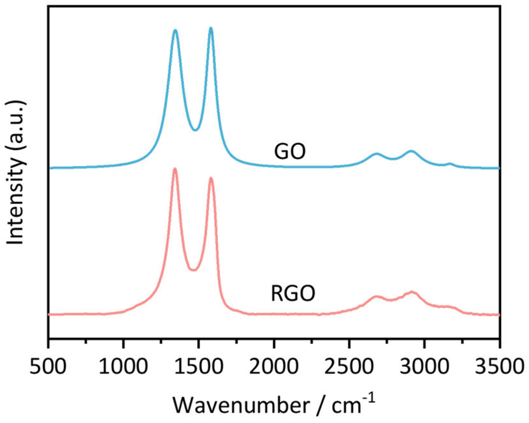

Figure S9. Raman spectra of GO and RGO. Two main broad D band and G band at 1340 and 1580 cm^{-1} were observed, respectively. The I_D/I_G ratio of RGO increased from 0.99 to 1.05 after solar reduction, indicating the restoration of sp^2 hybridized conjugated regions on RGO sheets.

1.10 Porosity of GF and PGF-(2-10)

Table S1. Porosity of GF and PGF-(2-10).

Sample	Porosity (%)
GF	98.4
PGF-2	96.8
PGF-4	94.5
PGF -6	91.8
PGF -8	86.1
PGF-10	80.3

1.11 Compressive Stress-Strain Curves of GF and PGF-(2-10)

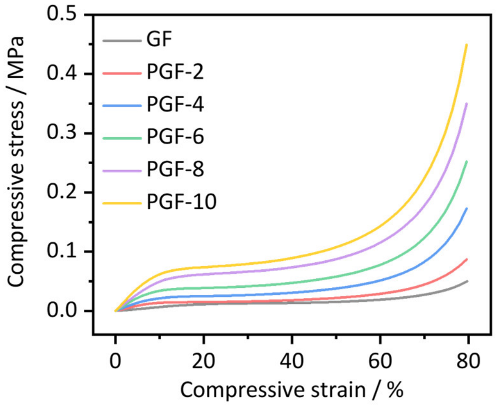

Figure S10. Compressive stress-strain curves of GF and PGF-(2-10) with the maximum strain of 80%.

1.12 Bending Stress-Strain Curves of GF and PGF-(2-10)

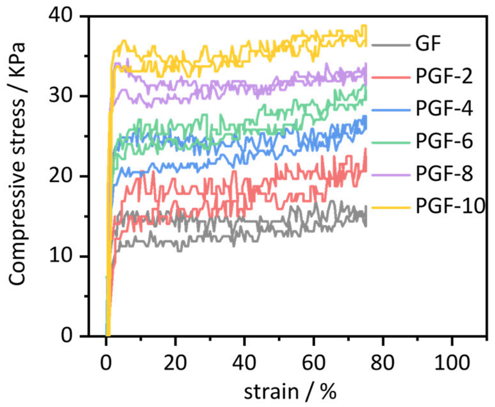

Figure S11. Bending stress-strain curves of GF and PGF-(2-10).

1.13 Tensile Stress-Strain Curves of GF and PGF-(2-10)

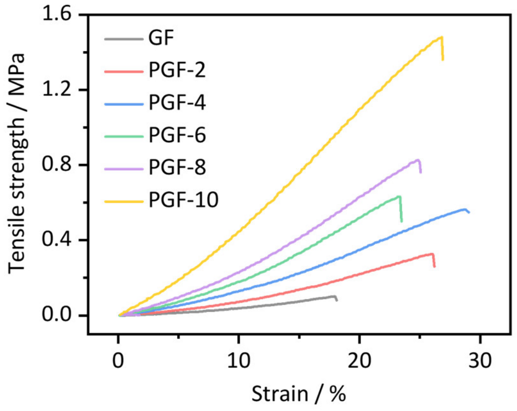

Figure S12. Tensile stress-strain curves of GF and PGF-(2-10).

1.14 The Durability of GF and PGF-(2-10) under Harsh Tests

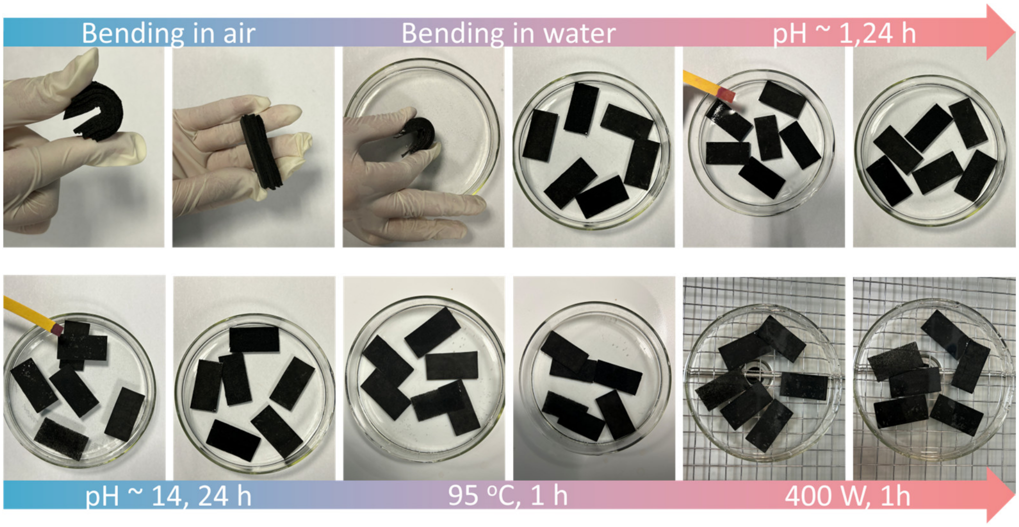

Figure S13. The durability and stability of the GF and PGF-(2-10) under a series of continuous harsh tests: 20 cycles of bending in air and water, acid (pH ~ 1, 24 hours), base (pH ~ 14, 24 hours), high temperature (~95 °C, 1 hour), and ultrasonic agitation (400 W, 1 hour).

1.15 SEM Image of the Graphene Sponge

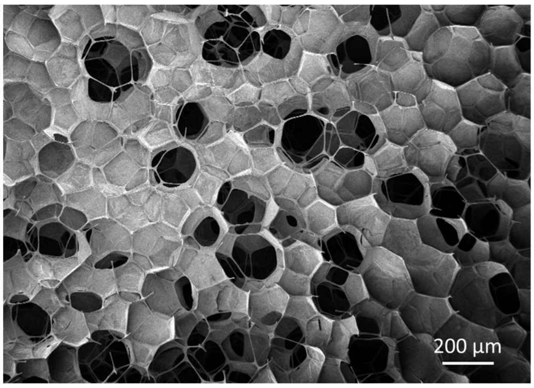

Figure S14. SEM image of the graphene sponge with random pores.

1.16 Stepwise Fabrication Process of the GG Sponge

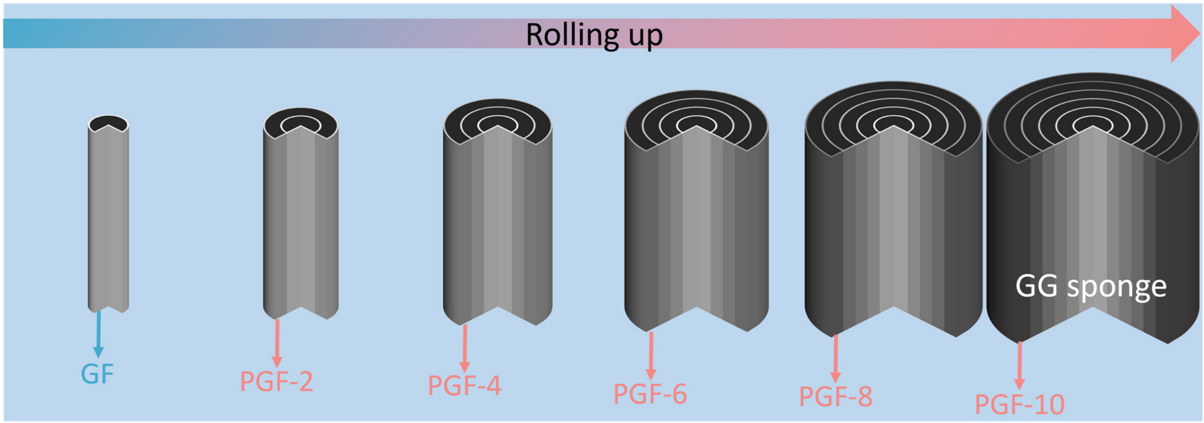

Figure S15. The stepwise fabrication process of the GG sponge.

1.17 Photograph of the GG Sponge after Continuous Solar Desalination

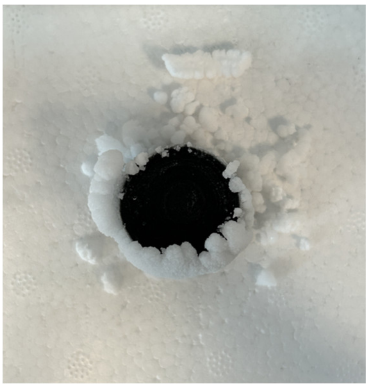

Figure S16. A top view of the GG sponge after a continuous 48-hour desalination in 20 wt% brine. The upper surface of the GG sponge was very clean with negligible salt accumulation.

1.18 Photograph of the GGS Sponge after Continuous Solar Desalination

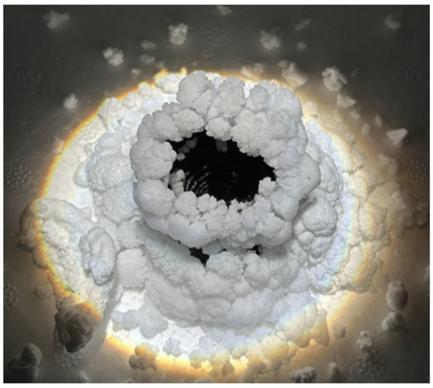

Figure S17. A top view of the GGS sponge after a continuous 48-hour desalination in 20 wt% brine. The inner side of the GGS sponge was very clean with negligible salt accumulation.

1.19 UV-vis-NIR spectra of GGS and GG Sponges

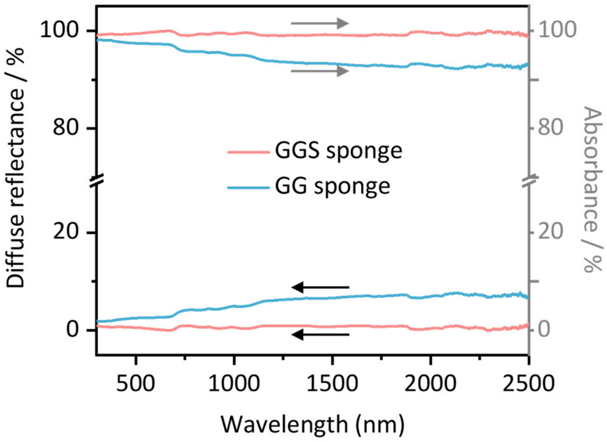

Figure S18. UV-vis-NIR reflectance and absorption spectra of wet GG and GGS sponges.

1.20 The Temperature Changes of GGS and GG Sponges under One-Sun Illumination

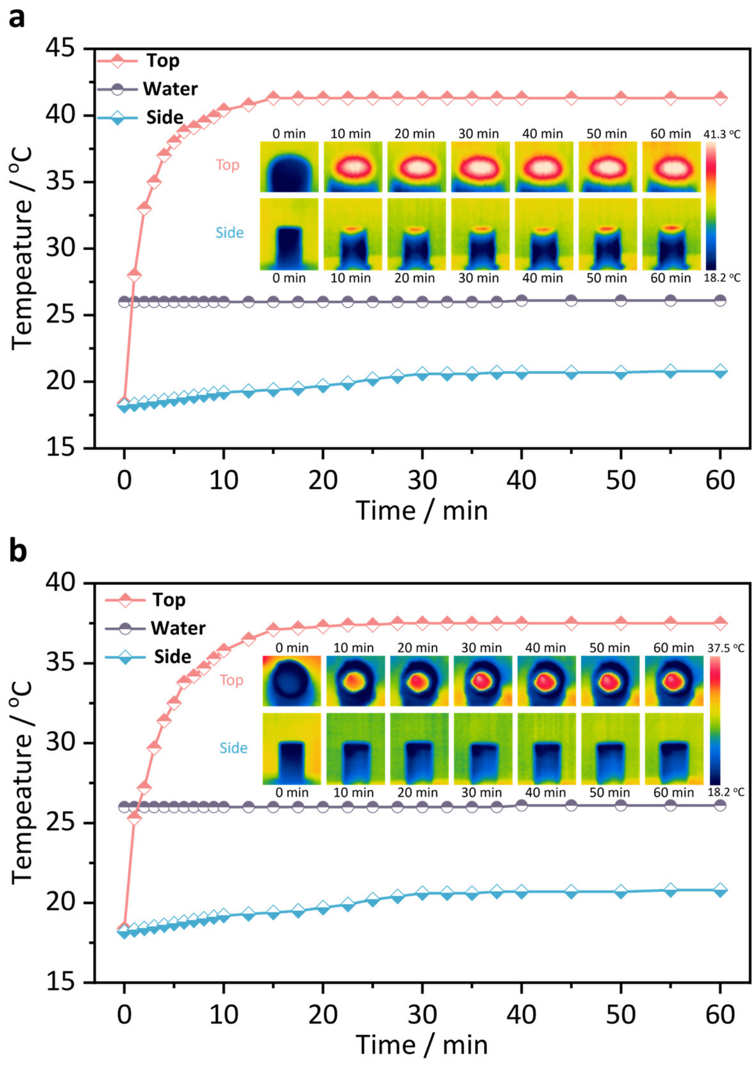

Figure S19. The temperature changes of the top and side surfaces of (a) GG and (b) GGS sponges as a function of time during solar desalination under one-sun irradiation. Inset: infrared images of the top and side surfaces.

1.21 Schematic Illustration for the Experimental Setup of the Solar Desalination

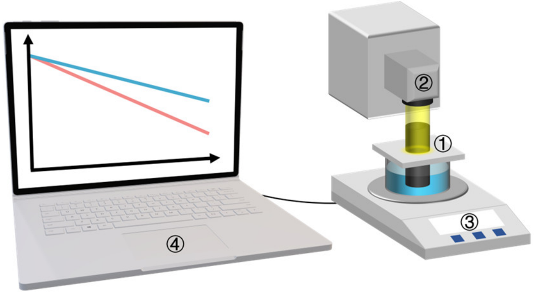

Figure S20. The scheme of the experiment setup for measuring solar desalination performance: ① GGS sponge, ② solar simulator, ③ electrical balance, ④ laptop.

1.22 The Comprehensive Solar Desalination Performance of the GGS Sponges with Other Solar Desalination Devices

Table S2. The comprehensive solar desalination performance of the GGS sponges compared with those of other previously reported solar desalination devices.

Sample	Average evaporation rate (kg m^{-2} h^{-1}; one sun)	Continuous evaporation time (h)	Salinity (%)	Ref
3D AGA IV equipped with p-MF	4.11	36	20	S1
Dual-zone photothermal sphere	2.60	8	3.5	S2
Dual-zone photothermal sphere	2.30	8	7	S2
Dual-zone photothermal sphere	2.06	8	20	S2
Hierarchically Designed Salt-Resistant Solar Evaporator	1.70	6	15	S3
Janus evaporator	1.35	6.7	10	S4
30° cone-shaped Janus evaporator	1.73	9	3.5	S5
2.5D Cu/CuO foam	4.05	4	3.5	S6
2.5D Cu/CuO foam	3.40	1	15	S6
Hanging PANi-cotton fabric	1.90	12	21	S7
Sponge-M-BHNPs	2.90	6	3.5	S8
Lily-inspired hierarchical structure	1.39	8	10	S9
Conic array assembly of Fe_3O_4@G nanoparticles	1.60	12	3.5	S10
Surface-carbonized bimodal porous wood membrane	0.80	7	15	S11
Hierarchically porous hydrogel	2.25	10	3.5	S12
PPy-coated glass fiber	1.15	10	10	S13
Janus 3D solar crystallizer	2.55	8	20	S14
Thermoelectricity-freshwater cogenerator	1.65	13	10	S15
Metal–phenolic networks	2.25	10	20	S16
The plasmonic wooden flower	1.60	12	1	S17
Acrylic ester resin/carbon nanofibers	1.60	5	20	S18
3D gel with vertical radiant structures	3.53	8	20	S19
Janus wood evaporator	1.20	8	20	S20
Anti-clogging graphite film	1.01	20	15	S21
a-MoC_{1-x}@CB	2.80	12	3.5	S22
Au@Ag-Pd/PS	2.84	40	3.5	S23
PANI/HPAN/CNTs nanofiber aeroge	3.00	50	3.5	S24
GGS sponge (4 cm)	4.7	72	20	This work

GGS sponge (6 cm)	6.5	72	20	This work
GGS sponge (8 cm)	7.5	72	20	This work

1.23 The Continuous Solar Desalination of the GGS Sponges with Different Heights

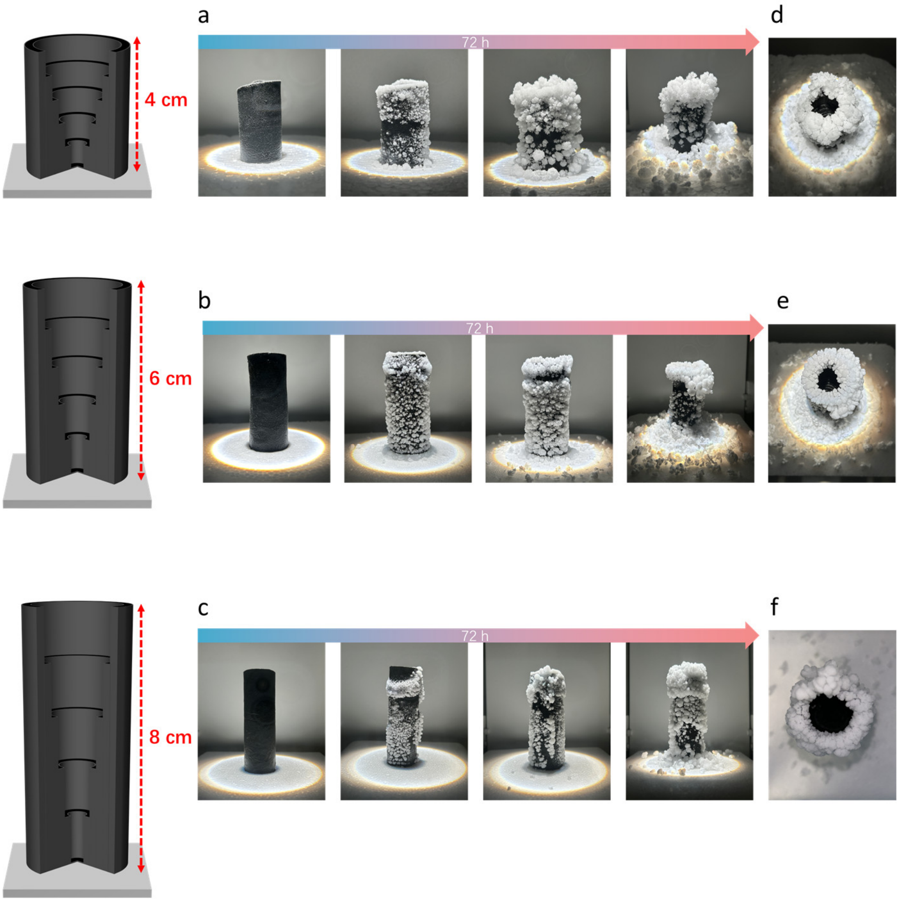

Figure S21. (a-c) Photographs of the GGS sponges (4, 6, and 8 cm) during continuous desalination tests under one sun illumination and (d-f) photographs of their inner side after a continuous 72 h desalination in 20 wt% brine, respectively.

1.24 The Salt Crystallization of the GGS Sponge Compared with Other Directional Crystallization Evaporators

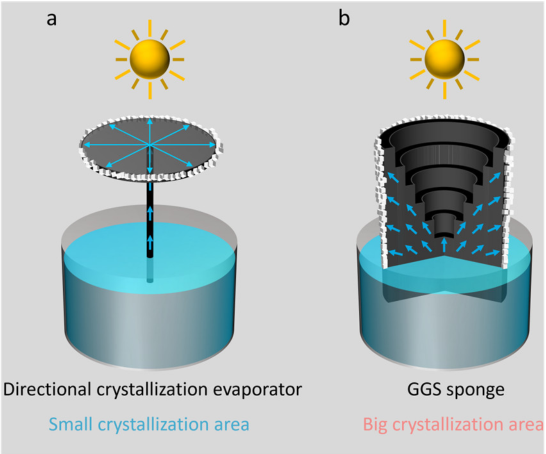

Figure S22. (a) Schematic illustration of edge-preferential salt crystallization of directional crystallization evaporators. Due to the thin evaporation disc, the area available for edge-preferential crystallization is limited. (b) Schematic illustration of directional crystallization of GGS sponge. The GGS sponge has the entire outer surface for salt crystallization, which provides a much larger crystallization area.

1.25 Salt Crystallization on the GGS Sponge during Continuous Solar Desalination

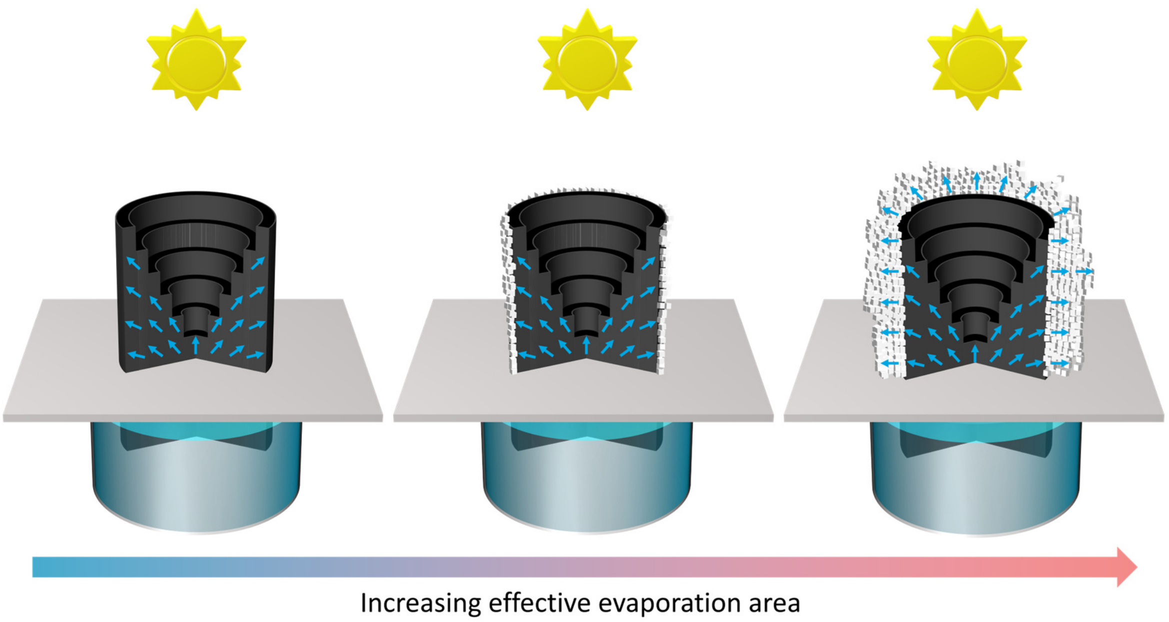

Figure S23. Salt crystallization on the GGS sponge during continuous solar desalination process.

1.26 Photo Images of the GGS Sponge after Removing Accumulated Salt

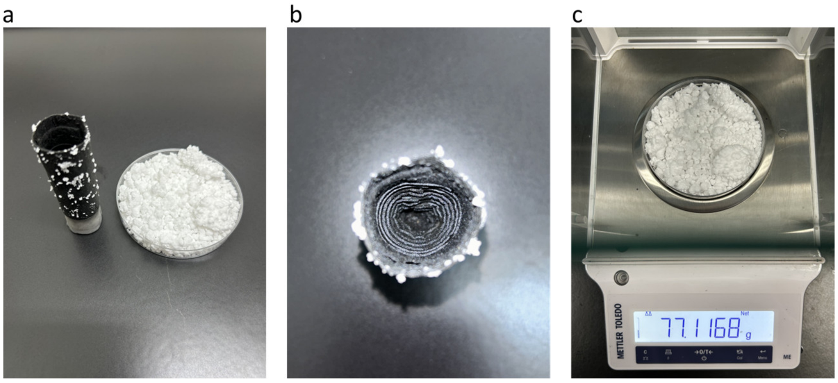

Figure S24. (a) Photo image of the GGS sponge after removing deposited salt from treating 20 wt.% brine. The coral-shape salt crystal layer could be easily removed by a stainless-steel medicine spoon. (b) After removing the salt deposited on the outer surface, the inner side of the GGS sponge was very clean with negligible salt observed. (c) Weight of salt collected.

1.27 Comparison of the Salt Collection Performance of the GGS Sponges and Other Solar Desalination Systems

Table S3. The salt collection rate of the GGS sponges compared with those of other previously reported solar evaporators.

Sample	Average evaporation rate ($kg\ m^{-2}\ h^{-1}$)	Salinity (%)	Salt collection rate ($kg\ m^{-2}\ h^{-1}$; one sun)	Ref
T-shaped synthetic tree	2.03	3.5	0.060	S25
T-shape C-L-wood evaporator	1.93	3.5	0.057	S26
Thermal radiation-enabled evaporation system	2.30	7	0.158	S27
PPy-GF	2.58	10	0.152	S13
Janus-based interfacial solar evaporator	1.21	10	0.037	S28
Janus evaporator	0.90	3.5	0.004	S29
Photovoltaicmultistage stiller	1.17	17	1.02	S30
3D AGA IV equipped with p-MF	4.11	20	0.370	S1
Co-NCNT-GO system	1.44	3.5	0.047	S31
Emerging solar-driven passive multistage distillation (MSD) devices	2.21	3.5	0.060	S32
Cu MOF-based nanorod arrays	1.78	3.5	0.047	S33
Carbon black PVA sponge	1.42	10	0.006	S34
3D cup shaped solar evaporator	1.33	25	0.342	S35
GGS sponge (4 cm)	4.7	20	1.1	This work
GGS sponge (6 cm)	6.5	20	1.5	This work
GGS sponge (8 cm)	7.5	20	1.8	This work

1.28 Photographs of the Water Collection Device in an Outdoor Desalination Test

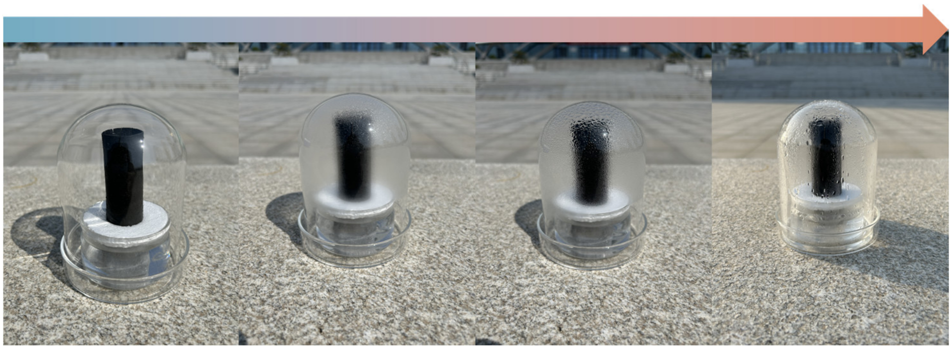

Figure S25. Photographs of the solar desalination device based on the GGS sponge for freshwater collection during an outdoor solar desalination test (from 9:00 AM to 5:00 PM on March 30, 2023, in Jinan).

1.29 The Environmental Conditions during the Long-Term Outdoor Solar Desalination Tests

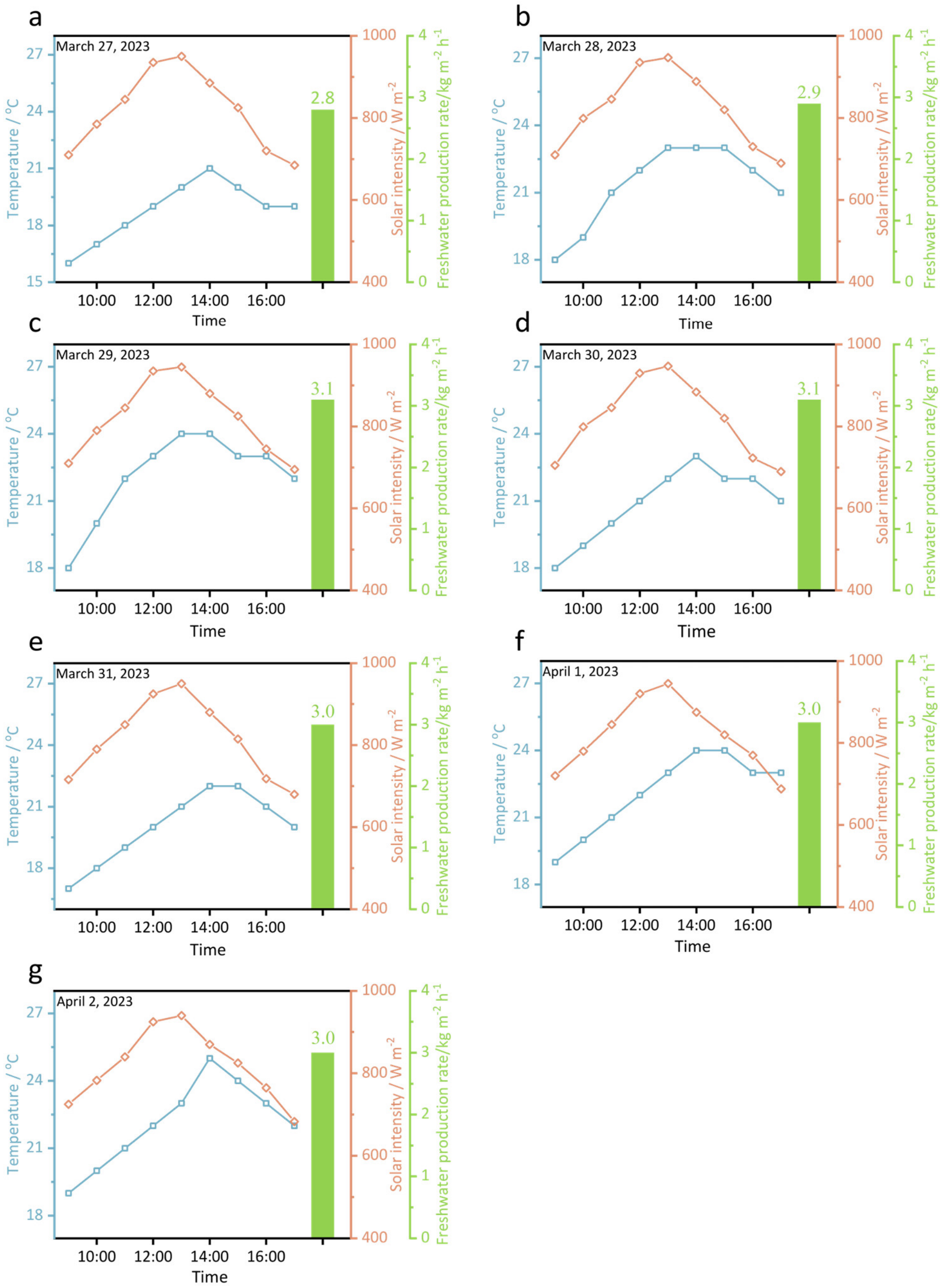

Figure S26. The environmental conditions (temperature and solar irradiation intensity) during the outdoor solar desalination tests from March 27 to April 2, 2023.

1.30 Water Production Performance of GGS Sponge and Other Solar Evaporation Devices Under Natural Sunlight Conditions

Table S4. The water production rate of the GGS sponge compared with those of other previously reported solar evaporation devices under natural sunlight conditions.

Sample	Solar intensity ($kW\ m^{-2}$)	Salinity (%)	Average water collection rate ($kg\ m^{-2}\ h^{-1}$)	Ref
melamine-urea-formaldehyde aerogel evaporator	Outdoor	3.5	1.57	S36
Co-NCNT/CF-based evaporator	Outdoor	0	1.50	S37
CFM@PDA hanging-model evaporator	Outdoor	0	1.13	S38
CFM@PDA hanging-model evaporator	Outdoor	3.5	0.48	S38
MXene/Au@Cu2−xS membrane	Outdoor	3.5	0.90	S39
Janus nano-micro structures	Outdoor	3.5	1.10	S23
nanofibrous aerogel evaporator	Outdoor	0	2.60	S24
polyzwitterionic hydrogel evaporators	Outdoor	3.5	0.77	S40
antifungal wooden cone evaporator	Outdoor	0	1.59	S41
wood-inspired bimodal evaporator	Outdoor	20	2.40	S42
turnover PPy-CF evaporator	Outdoor	3.5	0.90	S43
Polyaniline nanocone arrays evaporator	Outdoor	3.5	1.32	S44
Diode-like scalable asymmetric evaporator	Outdoor	3.5	0.89	S45
Metal-phenolic networks	Outdoor	10	1.82	S16
Metal-phenolic networks	Outdoor	15	1.76	S16
Metal-phenolic networks	Outdoor	20	1.65	S16
C@CuO-J60 Janus evaporator	Outdoor	3.5	1.53	S46
salt-resistant system	Outdoor	3.5	0.50	S47
waterbomb origami tower	Outdoor	3.5	1.22	S48
Cu NPs/LIG@ non-woven fabric evaporator	Outdoor	20	0.18	S49
Highly interconnected sponge	Outdoor	3.5	0.45	S50
GGS sponge	Outdoor	20	3.0	This work

1.31 Schematic Illustration of Salt Collection Salt by Replacing the Outer Layer of the GGS Sponge

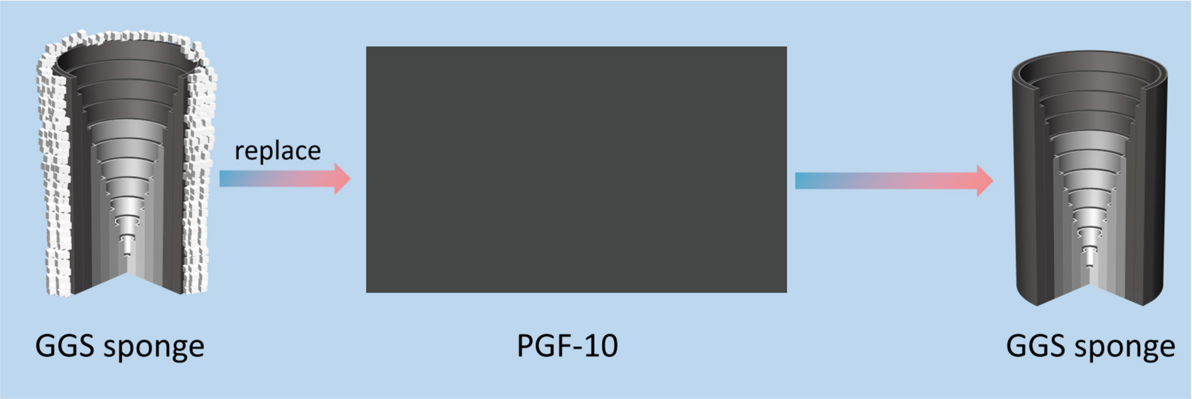

Figure S27. Salt collection by replacing the outer layer of the GGS sponge.

1.32 The Continuous Desalination Performance of the GGS Sponge and Other Solar Desalination Devices

Table S5. Comparison of continuous desalination performance of the GGS sponge and other solar evaporators.

Sample	Average evaporation rate (kg m^{-2} h^{-1}; one sun)	Continuous evaporation time(h)	Salinity (wt%)	Salt collection rate (kg m^{-2} h^{-1})	ZLD	Ref
Hierarchical graphene foam	1.40	1.5	2.75	/	No	S51
Hydrogel/rGO evaporator	2.50	96	10	/	No	S52
Plasmonic wood	0.95	8	3.5	/	No	S53
CB/PMMA-PAN	1.15	1	20	/	No	S54
Commercial absorber	0.94	10	3.5	/	Yes	S55
Surface-carbonized wood membrane	0.80	7	15	/	No	S11
Surface-carbonized wood membrane	0.82	100	1.1	/	No	S11
Filter paper/CNT	0.98	600	15	0.004	No	S29
Carbonized wood	1.04	100	20	/	No	S56
Coniform 3D evaporator	2.54	240	25	/	No	S57
Solar absorber/ blackbody emitter	0.49	3	25	/	Yes	S58
Bridge-Arch Evaporator	1.59	200	10	/	No	S18
Bridge-Arch Evaporator	1.60	5	20	/	No	S18
Janus wood evaporator	1.20	8	20	/	No	S20
Flower-like α-MoC1- x@CB evaporator	2.80	12	3.5	/	Yes	S22
Flower-like α-MoC1- x@CB evaporator	2.80	400	20	/	Yes	S22
2.5D Cu/CuO foam	4.05	4	3.5	/	No	S6
2.5D Cu/CuO foam	3.40	1	15	/	No	S6
3D gel with vertical radiant structures	3.53	8	20	/	No	S19
3D SE foam particles	2.09	100	15	/	No	S59
GGS sponge	6.6	144	20	1.50	Yes	This work

1.33 The Cost-Effectiveness of the GGS Sponge

Table S6. The total material cost and cost-effectiveness of the GGS sponge.

Materials	Single price ($ kg^{-1})	Single price ($ m^{-2})	Installation cost ($ m^{-2})	Total cost ($ m^{-2})	Cost-effectiveness (Rate/Price, g h^{-1} $$^{-1}$)
GO	260.30	--	39.46	53.87	126.23
CNCs	49.32	--	1.87		
MF	--	0.96	12.46		
NaCl	4.11	--	0.08		

1.34 The Cost-Effectiveness Comparison with Reported Evaporators

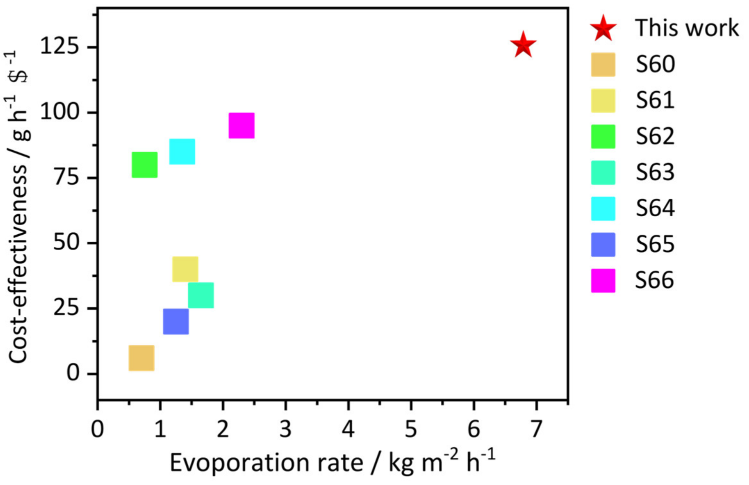

Figure S28. Comparison of the cost-effectiveness of the GGS sponge and other solar evaporators.

1.35 Schematic Illustration of Continuous Desalination-Irrigation Device

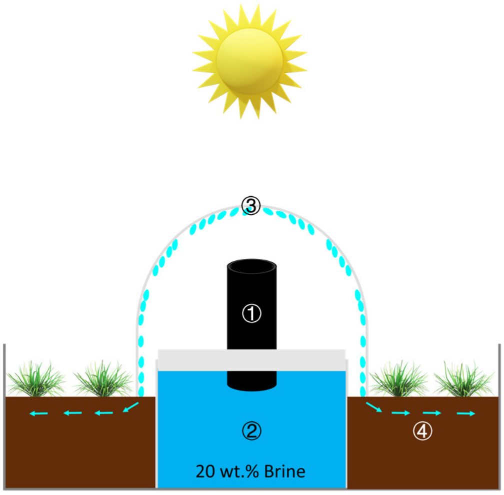

Figure S29. Schematic illustration of the continuous desalination-irrigation device based on the GGS sponge. This miniature desalination-irrigation device comprises four components: ① GGS sponge, ② a tank for 20 wt% brine, ③ a glass cover for condensation, and ④ soil.

1.36 Growth of Wheat Seedlings Irrigated with Different Water Sources

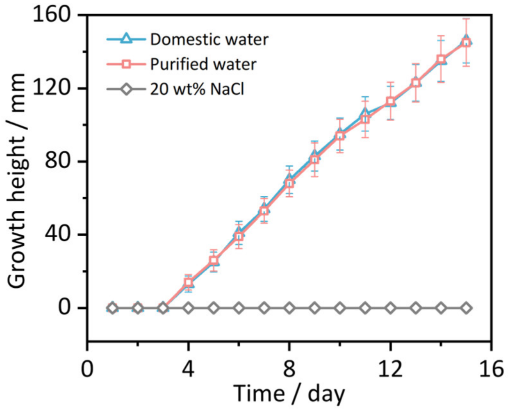

Figure S30. Growth height of wheat seedlings irrigated with different water sources over time.

1.37 Effect of 20 wt% Brine and Purified Water on Wheat Seed Germination

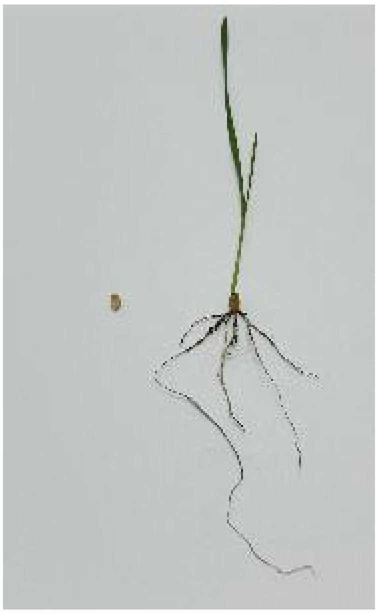

Figure S31. Comparison of germination of wheat seed treated with 20 wt% brine (left) and purified water (right).

2. Materials

Graphite powder was sourced from Qingdao Huagao Co., Ltd. (China). $KMnO_4$, concentrated H_2SO_4 (98%), concentrated hydrochloric acid (36-38%), and hydrogen peroxide (30%) were all obtained from Beijing Chemical Factory (China). The melamine foam (MF) was provided by Beiyou Company (Shanghai). A dialysis bag was purchased from Beijing Jingke Hongda Biotechnology Co., Ltd. (China). Seawater was collected from the Yellow Sea (Qingdao, China).

3. Methods

3.1 Preparation and Purification of GO

Graphene oxide (GO) was synthesized by a modified Hummer's method[S67]. The procedure was as follows: Natural graphite powder (2 g) was mixed with concentrated H_2SO_4 (50 mL) and stirred in an ice bath. $KMnO_4$ (6 g) was gradually added to the mixture under continuous stirring. The mixture was then transferred to a water bath at 35 °C and stirred for 30 min. Ultrapure water (200 mL) was slowly added to the mixture, followed by mechanical stirring for 24 h. The mixture was diluted and 5 mL of 30% H_2O_2 solution was dropwise added. The mixture was then centrifuged three times at 5000 rpm for 30 min in HCl solution (150 mL). The resulting wet solid was re-dispersed in ultrapure water (150 mL), centrifuged and washed three times with ultrapure water. Finally, the wet GO was dialyzed in ultrapure water for one week and sheared at high speed, and stored for future use.

3.2 Preparation of Cellulose Nanocrystals (CNCs)

Cotton linter pulp (CLP) was used as the main raw material to prepare cellulose nanocrystals (CNCs) by modified sulfuric acid hydrolysis. The procedure was as follows: Firstly, 5 g of ground CLP was soaked in a 4 wt % aqueous NaOH solution for 24 h at room temperature, which is called alkalization pretreatment. The resulting slurry was filtered, washed with

distilled water until the pH was neutral, and then dried in a vacuum oven at 60 °C for 24 h. Secondly, $CuSO_4$ (CLP / $CuSO_4$ = 100:1) was added to 64 wt % sulfuric acid solution to accelerate the hydrolysis reaction and improve the yield of CNCs. The CLP was dispersed into the acid solution at room temperature (CLP / acid = 1 g/16 mL) and stirred vigorously at 50 °C for 50 min, then the hydrolysis was stopped by diluting the mixture with cold water. The obtained suspension was centrifuged and washed until the pH ≈ 2. Finally, a milky-white suspension of CNCs was obtained. The CNCs had negatively charged sulfate ester groups on their surfaces, which made them stable in water dispersion.[S68]

3.3 Preparation of Pre-Pressed Porous Melamine Film (PMF)

Commercial MF was thoroughly washed with ethanol and deionized water. MFs with different thicknesses of 2, 4, 6, 8, and 10 mm were hot-pressed at ~230 °C in an oven to obtain the pre-pressed porous melamine films (PMFs) with a thickness of 1 mm, denoted as PMF-2, PMF-4, PMF-6, PMF-8, and PMF-10, respectively.

3.4 Preparation of Gradient Graphene Spiral Sponge (GGS Sponge)

A mixture of GO and CNCs was prepared by adding a CNCs suspension of 6 mg mL^{-1} to a GO solution of 6 mg mL^{-1} with a volume ratio of 4:1 and stirring vigorously. The MF and PMF- (2-10) with a thickness of 1 mm and increasing widths were continuously processed in three steps: spray coating, air-drying, and solar reduction. The GO/CNCs mixed suspension was coated on the MF and PMF-(2-10) by a simple spray method. The composites were then air-dried at 60 °C for 1 h. Next, the GO/CNCs/MF and GO/CNCs/PMF-(2-10) composites were exposed to focused sunlight irradiation for rapid reduction of GO to RGO. Finally, RGO/CNCs/MF (GF) and RGO/CNCs/PMF-(2-10) (PGF-(2-10)) were sequentially rolled, forming a gradient graphene spiral sponge (GGS sponge) with a pore size ranging from ~150 μm to ~20 μm. The resulting GGS sponges have inner heights of 4, 6, and 8 cm and a diameter of 2.7 cm.

3.5 Preparation of Gradient Graphene Sponge (GG Sponge)

Based on the MF and PMF-(2-10) with a thickness of 1 mm and the same width, the gradient graphene sponge (GG sponge) was prepared by the method described in section 3.4 (Preparation of Gradient Graphene Spiral Sponge (GGS Sponge)).

4. Physical Characterizations and Measurement

4.1 Characterizations

SEM images were obtained on a field-emission scanning electron microscope (Supra 55, ZEISS, Germany) using an accelerating voltage of 10 kV. All samples were spray-coated with a thin gold layer in vacuum prior to the SEM observations. Atomic force microscopy (AFM) images were recorded under ambient conditions using a Digital Instrument Multimode Nanoscope IIIA operating at a tapping mode. Transmission electron microscopy (TEM) was performed on a JEOL JEM-2010. X-ray diffraction (XRD) patterns were recorded using an X´Pert-ProMPD (Holland) D/max-γA X-ray diffractometer with Cu-Kα radiation (λ=0.154056 nm) with a scanning rate of 5° min^{-1} for 2 θ from 5° to 50°. X-ray photoelectron spectroscopy (XPS) measurements were carried out with an ESCALab220i-XL photoelectron spectrometer from VG Scientific. Raman spectroscopy was measured on a laser Raman microscope (inVia Reflex, Renishaw, England). Mercury intrusion porosimetry analysis was performed on a Quantachrome PoreMaster 60 instrument (Anton Paar, USA). Before the measurements, all samples were dried in vacuum at 50 °C for 12 h. The compressive tests were performed with a rheometer (ARES-G2, TA Instruments, USA) using a 50 N load cell in the axial-compression testing mode at a strain rate of 10 mm min^{-1}. The bending test was performed with a rheometer (ARES-G2, TA Instruments, USA) in the axial-tensile testing mode at rate of 0.5 mm s^{-1}.The tensile test was performed with a rheometer (ARES-G2, TA Instruments, USA) in the axial-tensile testing mode at rate of 0.5 mm s^{-1}. The optical transmittance (T) and reflectance (R) spectra were measured in the range of 300 - 2500 nm

with a spectrophotometer (UV3600, Shimadzu, Japan) attached to an integrating sphere (ISR-3100). The absorption efficiency is calculated by A=1-R-T, where R and T are the reflection efficiency and the transmission efficiency, respectively.

4.2 Evaluation of Solar Desalination in Laboratory

Solar simulator (CEL-S500-T5) with an optical filter for the standard AM 1.5 G spectrum was used to simulate sunlight. The environmental temperature was ~26 °C and the relative humidity was ~ 30%. We use the top projected plane of sample for the light intensity measurement and the radiation intensity is corrected by an optical power meter. The size of the light spot was controlled by an aperture, ensuring that it is equal to the size of the sample. Each sample was continuously illuminated for 1 h (solar desalination test) or 72 h (countinuous solar desalination test) or 144 h (long-term stability test) under simulated solar irradiation of 1 kW m^{-2}, and the mass loss was monitored in real-time with an electronic balance (FA 2004, 0.1 mg in accuracy). We calculated the evaporation rate by dividing the mass loss of the sample by its evaporation time and projected area.[S14,S16,S25,S29,S35] An IR camera (FLIR E5xt) was utilized to measure and record the temperature changes during solar water evaporation solar desalination. To prevent the direct evaporation of the underlying bulk brine from affecting the experimental results, we cut a 2.7 cm-diameter circular hole in the center of the polyethylene foam and inserted the sample into it.

4.3 Thermal Loss Analysis

One of the main challenges in solar evaporation is to minimize heat loss. In this work, we calculated the heat loss in detail and found that using radiative heat by designing the evaporator properly can effectively use the heat to speed up the water evaporation rate. The main types of heat loss are: conduction, convection, and radiation, as shown in Figure S32.

The following equations are used to calculate the heat losses due to conduction, convection, and radiation, respectively.[S61]

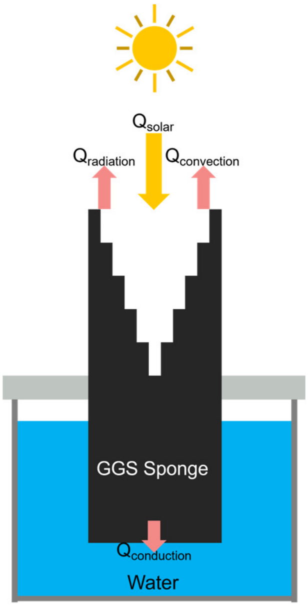

Figure S32. Thermal environment and heat transfer diagram of a solar evaporator. The red arrows represent the heat loss from the evaporator. The yellow arrow represents the solar energy inputted to the evaporator.

a) Conduction

The conduction loss is calculated by the following equation:

$$Q_{\text{conduction}} = \frac{mC\Delta T_e}{At} \tag{1}$$

where m is the water mass in the container, C is the water's specific heat capacity, ΔT_e is the increase in water temperature within 60 minutes, A is the cross-sectional area, and t is time. As shown in Table S7, the conduction loss of both the GG sponge and the GGS sponge was 1.5% of the input heat flux (1000 Wm^{-2}), as they had the same increase in the bulk water temperature.

Table S7. Conduction heat loss out of input heat flux.

Materials	*m* (g)	*C* (J g^{-1} K^{-1})	ΔT_e (K)	*A* (cm^2)	Heat loss (%)
GG sponge	75.0	4.2	0.1	5.7	1.5
GGS sponge	75.0	4.2	0.1	5.7	1.5

b) Convection

The convection heat loss was estimated by Newton's law of cooling as follows:

$$Q_{\text{convection}} = h\Delta T \quad (2)$$

Where $Q_{\text{convection}}$ is the heat flux of the convective heat loss, h is the heat transfer co-efficient of air (5 W m^{-2} K^{-1}), and ΔT is the temperature difference between the center of the top surface of the photothermal material and the ambient temperature. The top surface temperatures of the GG sponge and GGS sponge are 41.3 °C and 32.0 °C, respectively, and the ambient temperature is 26.0 °C. According to the equation (2), the convective loss of the GG sponge is 7.7 % of the input heat flux, while the convective loss of the GGS sponge is 3.0 % (Table S8). Due to the small temperature difference, the convective loss in the GGS sponge is lower than that in the GG sponge.

Table S8. Convection heat loss out of input heat flux.

Materials	*h* (W m^{-2} K^{-1})	ΔT_e (K)	Heat loss (%)
GG sponge	5	15.3	7.7
GGS sponge	5	6.0	3.0

c) Radiation

The radiation loss from the evaporator to the environment and radiation energy exchange within the solar evaporator were calculated by Stefan Boltzmann's formula as follows:

$Q_{\text{radiation}} = \varepsilon\sigma(T_1^4 - T_2^4)$; radiative heat loss (3)

$Q_{\text{radiation}} = \varepsilon\sigma F(T_1^4 - T_2^4)$; radiative heat exchange within the solar evaporator (4)

Where ε is the emissivity, which is assumed to have a maximum value of 1 for the water evaporation process, σ is the Stefan Boltzmann's constant (5.68×10^{-8} W m^{-2} K^{-4}), T_1 is the surface temperature of the evaporator after solar evaporation under solar illumination, T_2 is

the ambient temperature above the absorber, and F is the shape factor (i.e., the fraction of radiative energy that is diffused from one surface and directly strikes the other surface without any intervening reflection).[S69] The shape factor is a key parameter for calculating the radiation heat exchange between surfaces. It depends entirely on the geometry of the radiating surface. We considered the shape factor for the calculation of the radiation heat exchange, because the GGS sponge can be simplified into a V-shaped structure (Figure S32).

Flat surface

For a flat surface, the shape factor (F_{11}) is zero. This means that the supporter does not receive any radiative heat energy from itself. All the radiative heat energy is dissipated to the environment.

V-shaped surface

We used the following equation to calculate the view factor for a V-shaped surface.[S69]

$\sum_{j=1}^{n} F_{ij} = 1$, j =1 to n; summability rule (5)

$A_i F_{ij} = A_j F_{ji}$; reciprocity relation (6)

$F_{11} + F_{12} = 1$ (7)

$A_1 F_{12} = A_2 F_{21}$ (8)

$F_{21} + F_{22} = 1$ (9)

$F_{21} = 1 - F_{22} = 1 - 0 = 1$ ($F_{22} = 0$; as it is flat surface) (10)

From equation (8) $F_{12} = (A_2/A_1)$ (11)

From equation (7) $F_{11} = 1 - (A_2/A_1)$ (12)

$F_{11} = 1 - \frac{d}{\sqrt{d^2+4H^2}}$ (13)

$F_{12} = 1 - F_{11}$ (14)

Therefore, $Q_{radiation}$ (radiation heat loss) = $\varepsilon\sigma F_{12}(T_1^4 - T_2^4)$ (15)

$Q_{radiation}$ (radiation heat energy exchange) = $\varepsilon\sigma F_{11}(T_1^4 - T_2^4)$ (16)

The surface temperatures of the GG sponge and GGS sponge are 41.3 °C and 37.5 °C,

respectively, while the ambient temperature is 26 °C. The GGS sponge had a diameter of 2.7 cm and a V-shaped depth (H) of 4.0 cm. Using the above equations, we calculated that the surface radiation heat loss of the GG sponge accounted for 10.0 % of the input heat flux. Since the surface of the GG sponge was flat, it did not utilize any additional radiation energy (Figure 3c). For the GGS sponge, the surface radiation heat loss was about 7.5 %. More importantly, the GGS sponge utilized part of the radiation heat (5.1 %) by transferring it to the thin liquid film on the V-shaped surface through reflection, instead of releasing it to the environment separately by radiation and convection (Figure 3e and Table S9). This unique strategy effectively reused the radiation heat, achieved energy recovery and minimized energy loss, and further enhanced the evaporation rate (Table S10).

Table S9. Radiative heat loss and gain out of input heat flux.

Materials	ε	σ (W m^{-2} K^{-4})	F_{11}	F_{12}	$\sigma (T_1^4 - T_2^4)$ (W m^{-2})	Heat loss (%)	Heat gain (%)
GG sponge	1	5.68×10^{-8}	0	-	100.45	10.0	0
GGS sponge	1	5.68×10^{-8}	0.68	0.32	74.77	7.5	5.1

Table S10. Total heat loss out of input heat flux.

Materials	Conduction loss (%)	Convection loss (%)	Radiation loss (%)	Total (%)
GG sponge	1.5	7.7	10.0	19.2
GGS sponge	1.5	3.0	2.4	6.9

4.4 Numerical Simulation

We used COMSOL Multiphysics software (5.6 Version) to simulate the water transport, heat transfer, salinity in brine, and salt crystallization mechanism during the evaporation process. We built a two-dimensional axisymmetric model of a coiled porous graphene structure for this numerical simulation.

Water transport

According to the continuity equation, the water flux entering the evaporator equals the water flux leaving the evaporator due to evaporation in the sustainable evaporation process. Assuming that the pores contain saline water with 20% concentration, we applied the flow

simulation based on the saturated flow theory. The spontaneous upward flow in the evaporator is driven by gravity and capillary force, and we simulated the brine transport by solving the following equations:

$$\nabla(\rho_w u_g) + \nabla(\rho_w u_c) = g_{evap} M_w \quad (17)$$

where ρ_w is the brine density, u_g and u_c are the velocities influenced by gravity and capillary force, respectively, and M_w is the molar mass of brine. The formula for u is:

$$u = -\frac{\mu}{\emptyset}\nabla p \quad (18)$$

where μ is dynamic viscosity of water, $\emptyset$ is porosity of the structure, and ∇p is the pressure gradient corresponding to gravity or capillary force.

Salt crystallization during evaporation

The salt concentration increases due to water loss during evaporation. Therefore, we conducted the simulation based on the water content. The brine transport in the evaporator is expressed by:

$$\nabla(\rho_w u_{wc} + \rho_w u_{wd}) = g_{evap} M_w \quad (19)$$

where u_{wc} is the convection velocity and u_{wd} is the diffusion velocity.

4.5 Outdoor Solar Desalination and Water Collection

A simple prototype of outdoor solar desalination devices based on a GGS sponge is demonstrated for land application. The GGS sponge (diameter 2.7 cm and height 6 cm) was enclosed by a transparent glass cover in an inner chamber containing 20 wt.% brine. The outdoor experiment for solar desalination was performed from 09:00 AM to 5:00 PM on the playground at Shandong First Medical University (Huaiyin District, Jinan, China) from March 27 to April 2, 2023. The mercurial thermometer and optical power meter monitored ambient air temperature and sunlight intensity, respectively. During the solar desalination, water vapor condensed continuously on the inner walls of the glass cover, forming freshwater

that flowed into the bottom container for collection. Over a week, the GGS sponge-based desalination device produced freshwater at high and stable rates of 2.7-3.1 kg m^{-2} h^{-1}.

4.6 Measurement of Ion Concentration

The ion concentrations of gathered freshwater were measured by the inductively coupled plasma emission spectrometer (ICPE-9820, Shimadzu, Japan).

4.7 Evaluation of Cost-Effectiveness

The cost-effectiveness ϕ is defined as $\phi = v/c$, where v represents the evaporation rate and c represents the material cost. The ϕ value indicates the amount of purified water that can be produced in 1 hour with 1 dollar.[S70]

4.8 Wheat Irrigation Process

Select three Petri dishes with a height of 2.5 cm and a diameter of 13 cm and fill them with natural soil with a height of 2 cm. Add 20 wt% brine, purified water, and domestic water to each Petri dish, respectively, to moisten the soil. Then evenly sprinkle 50 wheat seeds on the edge of each Petri dish and cover them with soil. Place the Petri dishes outside the experimental building door for the whole day. One of the Petri dishes is supplied with purified water every day by a desalination-irrigation device, while the other two Petri dishes are each added with 20 ml of domestic water and 20 wt% brine every day.

5. Reference

[S1] M. C. Ding, H. Lu, Y. B. Sun, Y. J. He, J. H. Yu, H. J. Kong, C. X. Shao, C.-Y. Liu, C. W. Li, Superelastic 3D assembled clay/graphene aerogels for continuous solar desalination and oil/organic solvent absorption. *Adv. Sci.* **2022**, 9, 2205202.

[S2] X. Wu, Y. D. Wang, P. Wu, J. Y. Zhao, Y. Lu, X. F. Yang, H. L. Xu, Dual-zone photothermal evaporator for antisalt accumulation and highly efficient solar steam generation. *Adv. Funct. Mater.* **2021**, *31*, 2102618.

[S3] W. Zhao, H. Gong, Y. Song, B. Li, N. Xu, X. Min, G. Liu, B. Zhu, L. Zhou, X. X. Zhang, J. Zhu, Hierarchically designed salt-resistant solar evaporator based on donnan effect for stable and high-performance brine treatment. *Adv. Funct. Mater.* **2021**, *31*, 2100025.
[S4] J. X. Chen, J. L. Yin, B. Li, Z. Y. Ye, D. L. Liu, D. Ding, F. Qian, N. V. Myung, Q. Zhang, Y. D. Yin, Janus evaporators with self-recovering hydrophobicity for salt-rejecting interfacial solar desalination. *ACS Nano* **2020**, *14*, 17419.
[S5] N. N. Cao, S. T. Lu, R. Yao, C. X. Liu, Q. Y. Xiong, W. Qin, X. H. Wu, A self-regenerating air-laid paper wrapped ASA 3D cone-shaped Janus evaporator for efficient and stable solar desalination. *Chemical Engineering Journal* **2020**, *397*, 125522.
[S6] X. Liu, Y. Tian, F. Chen, A. Caratenuto, J. A. DeGiorgis, M. Elsonbaty, Y. Wan, R. Ahlgren, Y. Zheng, An easy-to-fabricate 2.5D evaporator for efficient solar desalination. *Adv. Funct. Mater.* **2021**, *31*, 2100911.
[S7] Z. X. Liu, B. H. Wu, B. Zhu, Z. G. Chen, M. F. Zhu, X. G. Liu, Continuously producing watersteam and concentrated brine from seawater by hanging photothermal fabrics under sunlight. *Adv. Funct. Mater.* **2019**, *29*, 1905485.
[S8] Z. X. Wang, X. C. Wu, F. He, S. Q. Peng, Y. X. Li, Confinement capillarity of thin coating for boosting solar-driven water evaporation. *Adv. Funct. Mater.* **2021**, *31*, 2011114.
[S9] N. Xu, J. L. Li, Y. Wang, C. Fang, X. Q. Li, Y. X. Wang, L. Zhou, B. Zhu, Z. Wu, S. N. Zhu, J. Zhu, A water lily-inspired hierarchical design for stable and efficient solar evaporation of high-salinity brine. *Sci. Adv.* **2019**, *5*, eaaw7013.
[S10] Y. J. Hu, H. Y. Ma, M. M. Wu, T. Y. Lin, H. Z. Yao, F. Liu, H. H. Cheng, L. T. Qu, A reconfigurable and magnetically responsive assembly for dynamic solar steam generation. *Nat. Comm.* **2022**, *13*, 4335.
[S11] S. He, C. Chen, Y. Kuang, R. Mi, Y. Liu, Y. Pei, W. Kong, W. Gan, H. Xie, E. Hitz, C. Jia, X. Chen, A. Gong, J. Liao, J. Li, Z. J. Ren, B. Yang, S. Das, L. Hu, Nature-inspired salt resistant bimodal porous solar evaporator for efficient and stable water desalination. *Energy Environ. Sci.* **2019**, *12*, 1558.
[S12] F. B. Zhu, L. Q. Wang, B. Demir, M. An, Z. L. Wu, J. Yin, R. Xiao, Q. Zheng, J. Qian, Accelerating solar desalination in brine through ion activated hierarchically porous polyion complex hydrogels. *Mater. Horiz.* **2020**, *7*, 3187-3195.
[S13] Z. Yu, S. N. Li, Y. Chen, X. T. Zhang, J. Y. Chu, Y. X. Zhang, S. C. Tan, Intensifying the co-production of vapor and salts by a one-way brine-flowing structure driven by solar irradiation or waste heat. *Desalination.* **2022**, *539*, 115942.
[S14] R. Gu, Z. Yu, Y. Sun, Y. Su, W. Wu and S. Cheng, Janus 3D solar crystallizer enabling

an eco-friendly zero liquid discharge of high-salinity concentrated seawater with antiscalant. *Desalination.* **2022**, *537*, 115862.
[S15] X. J. Mu, J. H. Zhou, P. F. Wang, H. Chen, T. T. Yang, S. S. Chen, L. Miao, T. Mori, A robust starch-polyacrylamide hydrogel with scavenging energy harvesting capacity for efficient solar thermoelectricity-freshwater cogeneration. *Energy Environ Sci.* **2022**, *15*, 3388-3399.
[S16] Z. X. Wang, J. Gao, J. J. Zhou, J. W. Gong, L. W. Shang, H. B. Ye, F. He S. Q. Peng, Z. X. Lin, Y. X. Li, F. Caruso, Engineering metal-phenolic networks for solar desalination with directional salt crystallization. *Adv. Mater.* **2023**, *35*, 2209015.
[S17] S. Chen, Z. Sun, W. Xiang, C. Shen, Z. Wang, X. Jia, J. Sun and C.-J. Liu, Plasmonic wooden flower for highly efficient solar vapor generation. *Nano Energy*, **2020**, *76*, 104998.
[S18] M. Zou, Y. Zhang, Z. Cai, C. Li, Z. Sun, C. Yu, Z. Dong, L. Wu, Y. Song, 3D printing a biomimetic bridge-arch solar evaporator for eliminating salt accumulation with desalination and agricultural applications. *Adv. Mater.* **2021**, *33*, 2102443.
[S19] X. H. Liu, F. X. Chen, Y. K. Li, H. J. Jiang, D. D. Mishra, F. Yu, Z. H. Chen, C. Q. Hu, Y. Chen, L. T. Qu, W. T. Zheng, 3D hydrogel evaporator with vertical radiant vessels breaking the trade-off between thermal localization and salt resistance for solar desalination of high-salinity. *Adv. Mater.* **2022**, 2203137.
[S20] X. Chen, S. He, M. M. Falinski, Y. Wang, T. Li, S. Zheng, D. Sun, J. Dai, Y. Bian, X. Zhu, J. Jiang, L. Hu, Z. J. Ren, Sustainable off-grid desalination of hypersaline waters using Janus wood evaporators. *Energy Environ. Sci.* **2021**, *14*, 5347.
[S21] V. Kashyap, A. Al-Bayati, S. M. Sajadi, P. Irajizad, S. H. Wang, H. Ghasemi, A flexible anti-clogging graphite film for scalable solar desalination by heat localization. *J. Mater. Chem. A* **2017**, *5*, 15227.
[S22] L. Zhu, L. Sun, H. Zhang, H. Aslan, Y. Sun, Y. Huang, F. Rosei, M. Yu, A solution to break the salt barrier for high-rate sustainable solar desalination. *Energy Environ. Sci.* **2021**, *14*, 2451.
[S23] Z. Y. Chen, J. Wang, H. J. Zhou, Z. M. Xie, L. Shao, A. Z. Chen, S. B. Wang, N. Jiang, Janus nano-micro structure-enabled coupling of photothermal conversion, heat localization and water supply for high-efficiency solar-driven interfacial evaporation. *Adv. Funct. Mater.* **2023,** 2303656.
[S24] W. J. Ma, T. Lu, W. X. Cao, R. H. Xiong, C. B. Huang, Bioinspired nanofibrous aerogel with vertically aligned channels for efficient water purification and salt-rejecting solar desalination. *Adv. Funct. Mater.* **2023,** *33*, 2214157.

[S25] Y. Shao, J. B. Tang, N. B. Li, T. Y. Sun, L. P. Yang, D. Chen, H. Zhi, D. J. Wang, H. Liu, G. B. Xue, Designing a bioinspired synthetic tree by unidirectional freezing for simultaneous solar steam generation and salt collection. *EcoMat* **2020**, *2*, e12018.

[S26] H. W. Liu, R. Z. Jin, S. C. Duan, Y. J. Ju, Z. Y. Wang, K. Yang, B. D. Wang, B. Wang, Y. G. Yao, F. J. Chen, Anisotropic evaporator with a t-shape design for high-performance solar-driven zero-liquid discharge. *Small* **2021**, *17*, 2100969.

[S27] Y. Bian, Z. H. Ye, G. Y. Zhao, K. Tang, Y. Teng, S. Chen, L. J. Zhao, X. Yuan, S. M. Zhu, J. D. Ye, H. Lu, Y. Yang, L. Fu, S. L. Gu, Enhanced contactless salt-collecting solar desalination. *ACS Appl. Mater. Interfaces* **2022**, *14*, 34151-34158.

[S28] R. N. Gu, Z. Yu, Y. Sun, P. F. Xie, Y. H. Li, S. N. Cheng, Enhancing stability of interfacial solar evaporator in high-salinity solutions by managing salt precipitation with Janus-based directional salt transfer structure. *Desalination* **2022**, *524*, 115470.

[S29] Y. Xia, Q. F. Hou, H. S. Jubaer, Y. Li, Y. Kang, S. Yuan, H. Y. Liu, M. W. Woo, L. Zhang, L. Gao, H. T. Wang, X. W. Zhang, Spatially isolating salt crystallisation from water evaporation for continuous solar steam generation and salt harvesting. *Energy Environ. Sci.* **2019**, *12*, 1840.

[S30] L. P. Yang, T. Y. Sun, J. B. Tang, Y. Shao, N. B. Li, A. Q. Shen, J. J. Chen, Y. F. Zhang, H. Liu, G. B. Xue, Photovoltaic-multistage desalination of hypersaline waters for simultaneous electricity, water and salt harvesting via automatic rinsing. *Nano Energy* **2021**, *87*, 106163.

[S31] Y. Kong, Y. Gao, Y. N. Shang, W. J. Kong, Y. F. Qi, S. Q. Wang, F. J. Yin, B. Y. Gao, S. G. Wang, Q. Y. Yue, Synergistic adjustment of water channels and light absorption pathways to co-generate salt collection and clean water production. *Science of the Total Environment* **2021**, *797*, 148912.

[S32] Y. H. Li, S. N. Cheng, Z. Yu, R. N. Gu, X. Y. He, Stable, zero liquid discharge, and highly efficient solar-driven multistage distillation device based on tree-inspired radial water transfer. *J. Clean. Prod.* **2022**, *375*, 134025.

[S33] X. Ma, X. Y. Wan, Z. Fang, Z. Y. Li, X. B. Wang, Y. Hu, M. Y. Dong, Z. Z. Ye, X. S. Peng, Orientational seawater transportation through Cu(TCNQ) nanorod arrays for efficient solar desalination and salt production. *Desalination* **2022**, *522*, 115399.

[S34] T. X. Zhang, J. X. Zhao, L. Liang, C. L. Guo, Constructing a solar evaporator with salt-collecting paper by stacking hydrophilic sponges for freshwater production and salt collection. *ACS Appl. Mater. Interfaces* **2022**, *14*, 668-676.

[S35] Y. Shi, C. L. Zhang, R. Y. Li, S. F. Zhuo, Y. Jin, L. Shi, S. Hong, J. Chang, C. S. Ong, P. Wang, Solar evaporator with controlled salt precipitation for zero liquid discharge desalination. *Environ. Sci. Technol.* **2018,** *52*, 11822-11830.

[S36] Z. Y. Gui, Z. Y. Yang, D. P. Xiang, Efficient solar water evaporation enabled by Ti_3O_5/Ti_4O_7-based melamine-urea–formaldehyde aerogel evaporators. *Chemical Engineering Journal* **2023**, *466*, 143055.

[S37] L. F. Cui, C. W. Ma, P. F. Wang, H. N. Che, H. L. Xu, Y. H. Ao, Rationally constructing a 3D bifunctional solar evaporator for high-performance water evaporation coupled with pollutants degradation. *Applied Catalysis B: Environmental* **2023,** *337*, 122988.

[S38] W. M. Chong, R. R. Meng, Z. X. Liu, Q. Y. Liu, J. J. Hu, B. Zhu, D. K. Macharia, Z. G. Chen, L. S. Zhang, Superhydrophilic polydopamine-modified carbon-fiber membrane with rapid seawater-transferring ability for constructing efficient hanging-model evaporator. *Advanced Fiber Materials* **2023,** *5*:1063–1075.

[S39] H. S. Kang, J. W. Zou, Y. Liu, L. Ma, J. R. Feng, Z. Y. Yu, X. B. Chen, S. J. Ding, L. Zhou, Q. Q. Wang, Synergistic effect of photothermal conversion in MXene/Au@$Cu_{2-x}S$ hybrids for efficient solar water evaporation. *Adv. Funct. Mater.* **2023,** 2303911.

[S40] B. L. Peng, Q. Q. Lyu, M. M. Li, S. Du, J. T. Zhu, L. B. Zhang, Phase-separated polyzwitterionic hydrogels with tunable sponge-like structures for stable solar steam generation. *Adv. Funct. Mater.* **2023,** *33*, 2214045.

[S41] M. H. Xie, P. Zhang, Y. Z, Cao, Y. T. Yan, Z. Wang, C. D. Jin, A three-dimensional antifungal wooden cone evaporator for highly efficient solar steam generation. *npj Clean Water* **2023**, 6:12.

[S42] Y. Chen, J. Yang, D. F. Zhang, S. Z. Wang, X. H. Jia, Y. Li, D. Shao, L. Feng, H. J. Song, S. C. Tang, A wood-inspired bimodal solar-driven evaporator for highly efficient and durable purification of high-salinity wastewater. *J. Mater. Chem. A,* **2023,** *11*, 2349–2359.

[S43] X. Zhong, Y. M. Wu, P. Zhang, Y. B. Chen, Y. S. Cai, W. M. Wang, X. Min, J. Xiong, M. Li, Turnover polypyrrole decorated cotton fabric based solar evaporator for cost-effective and steady desalination. *Journal of Cleaner Production* **2023,** *417*, 138088.

[S44] X. Zhao, X. T. Meng, H. Q. Zou, Z. H. Wang, Y. D. Du, Y. Shao, J. Qi, J. S. Qiu, Topographic manipulation of Graphene oxide by polyaniline nanocone arrays enables high-performance solar-driven water evaporation. *Adv. Funct. Mater.* **2023,** *33*, 2209207.

[S45] Y. W. Yang, H. X. Feng, W. X. Que, Y. Qiu, Y. Q. Li, L. Guo, Q. Li, A diode-like scalable asymmetric solar evaporator with ultra-high salt resistance. *Adv. Funct. Mater.* **2023,** *33*, 2210972.

[S46] L. L. Hou, N. Wang, L. J. Yu, J. C. Liu, S. C. Zhang, Z. M. Cui, S. Li, H. Li, X. F. Liu, L. Jiang, Y. Zhao, High-performance Janus solar evaporator for water purification with broad spectrum absorption and ultralow heat loss. *ACS Energy Lett.* **2023,** *8*, 553−564.

[S47] C. Y. Dang, H. Wang, Y. T. Cao, J. Shen, J. Zhang, L. T. Lv, G. Y. Xu, M. F. Zhu, Ultra salt-resistant solar desalination system via large-scale easy assembly of microstructural units. *Energy Environ. Sci.* **2022**, *15*, 5405–5414.

[S48] X. J. Liu, Y. P. Tian, F. Q. Chen, Y. Mu, A. Caratenuto, M. Minus, Y. Zheng, A waterbomb origami tower for convertible photothermal evaporation. *J. Mater. Chem. A* **2022,** *10*, 18657-18670.

[S49] D. Xu, H. Zhong, M. G. Li, S. S. To, L. Lu, Efficient plasmonic enhanced solar evaporation achieved by laser-assisted Cu/Graphene nanocomposite. *Carbon* **2023**, *204*, 231-237.

[S50] Z. Y. Mao, Y. X. Chen, G. Li, X. G. Duan, J. D. Shen, Y. C. Han, L. X. Li, Z. Chen, H. Liu, J. Lu, Highly interconnected sponge with optimized water absorption and thermal conductivity for efficient solar desalination. *Separation and Purification Technology* **2023**, *314*, 123502.

[S51] H. Ren, M. Tang, B. Guan, K. Wang, J. Yang, F. Wang, M. Wang, J. Shan, Z. Chen, D. Wei, H. Peng, Z. Liu, Hierarchical Graphene foam for efficient omnidirectional solar–thermal energy conversion. *Adv. Mater.* **2017**, *29*, 1702590.

[S52] X. Zhou, F. Zhao, Y. Guo, Y. Zhang, G. Yu, A hydrogel-based antifouling solar evaporator for highly efficient water desalination. *Energy Environ. Sci.* **2018**, *11*, 1985.

[S53] M. Zhu, Y. Li, F. Chen, X. Zhu, J. Dai, Y. Li, Z. Yang, X. Yan, J. Song, Y. Wang, E. Hitz, W. Luo, M. Lu, B. Yang, L. Hu, Plasmonic wood for high-efficiency solar steam generation. *Adv. Energy Mater.* **2018**, *8*, 1701028.

[S54] W. Xu, X. Hu, S. Zhuang, Y. Wang, X. Li, L. Zhou, S. Zhu, J. Zhu, Flexible and salt resistant Janus absorbers by electrospinning for stable and efficient solar desalination. *Adv. Energy Mater.* **2018**, *8*, 1702884.

[S55] T. A. Cooper, S. H. Zandavi, G. W. Ni, Y. Tsurimaki, Y. Huang, S. V. Boriskina, G. Chen, Contactless steam generation and superheating under one sun illumination. *Nat. Commun.* **2018**, *9*, 5086.

[S56] Y. Kuang, C. Chen, S. He, E. M. Hitz, Y. Wang, W. Gan, R. Mi, L. Hu, A high-performance self-regenerating solar evaporator for continuous water desalination. *Adv. Mater.* **2019**, *31*, 1900498.

[S57] L. Wu, Z. Dong, Z. Cai, T. Ganapathy, N. X. Fang, C. Li, C. Yu, Y. Zhang, Y. Song, Highly efficient three-dimensional solar evaporator for high salinity desalination by localized crystallization. *Nat. Commun*. **2020**, *11*, 521.

[S58] A. K. Menon, I. Haechler, S. Kaur, S. Lubner, R. S. Prasher, Enhanced solar evaporation using a photo-thermal umbrella for wastewater management. *Nat Sustain.* **2020**, *3*, 144.

[S59] H. W. Liu, B. C. Chen, Y. L. Chen, M. N. Zhou, F. W. Tian, Y. Z. Li, J. J. Jiang, W. T. Zhai, Bioinspired self-standing, self-floating 3D solar evaporators breaking the trade-off between salt cycle and heat localization for continuous seawater desalination. *Adv. Mater.* **2023**, *35*, 2301596.

[S60] K. Bae, G. Kang, S. K. Cho, W. Park, K. Kim, W. J. Padilla, Flexible thin-film black gold membranes with ultrabroadband plasmonic nanofocusing for efficient solar vapour generation, *Nat. Commun.* **2015**, *6*, 10103.

[S61] X. Q. Li, W. C. Xu, M. Y. Tang, L. Zhou, B. Zhu, S. N. Zhu, J. Zhu, Graphene oxide based efficient and scalable solar desalination under one sun with a confined 2D water path, *Proc. Natl. Acad. Sci. U. S. A.* **2016**, *113*, 13953.

[S62] C. Finnerty, L. Zhang, D. L. Sedlak, K. L. Nelson, B. X. Mi, Synthetic graphene oxide leaf for solar desalination with zero liquid discharge, *Environ. Sci. Technol.* **2017**, *51*, 11701.

[S63] R. Hu, J. Q. Zhang, Y. D. Kuang, K. B. Wang, X. Y. Cai, Z. Q. Fang, W. Q. Huang, G. Chen, Z. X. Wang, A Janus evaporator with low tortuosity for long-term solar desalination, *J. Mater. Chem. A* **2019**, *7*, 15333.

[S64] J. Liu, J. H. Yao, Y. Yuan, Q. L. Liu, W. Zhang, X. H. Zhang, J. J. Gu, Surface carbonized bamboos with multilevel functional biostructures deliver high photothermal water evaporation performance, *Adv. Sustain. Syst.* **2020**, *4*, 2000126.

[S65] B. W. Lv, C. Gao, Y. L. Xu, X. F. Fan, J. K. Xiao, Y. M. Liu, C. W. Song, A self-floating, salt-resistant 3D Janus radish-based evaporator for highly efficient solar desalination, *Desalination* **2021**, *510*, 115093.

[S66] X. Q. Li, J. L. Li, J. Y. Lu, N. Xu, C. L. Chen, X. Z. Min, B. Zhu, H. X. Li, L. Zhou, S. N. Zhu, T. J. Zhang, J. Zhu, Enhancement of interfacial solar vapor generation by environmental energy, *Joule* **2018**, *2*, 1331.

[S67] B. Huo, D. Jiang, X. Cao, H. Liang, Z. Liu, C. Li, J. Liu, N-doped graphene /carbon hybrid aerogels for efficient solar steam generation. *Carbon* **2019**, *142*, 13.

[S68] X. Zhang, P. Liu, Y. Duan, M. Jiang, J. Zhang, Graphene/cellulose nanocrystals hybrid aerogel with tunable mechanical strength and hydrophilicity fabricated by ambient pressure drying technique. *RSC Adv.* **2017**, *7*, 16467.

[S69] A. Mezrhab, M. Bouzidi, Eng. Comput. (Swansea, Wales) **2005**, *22*, 132.

[S70] Z. X. Wang, X. C. Wu, J. M. Dong, X. H. Yang, F. He, S. Q. Peng, Y. X. Li, Porifera-inspired cost-effective and scalable "porous hydrogel sponge" for durable and highly efficient solar-driven desalination, *Chem. Eng. J.* **2022**, *427*, 130905.
